# Global burden of type 1 diabetes in adults aged 65 years and older, 1990-2019: population based study

**DOI:** 10.1136/bmj-2023-078432

**Published:** 2024-06-12

**Authors:** Kaijie Yang, Xue Yang, Chenye Jin, Shuangning Ding, Tingting Liu, Bing Ma, Hao Sun, Jing Zhang, Yongze Li

**Affiliations:** 1 Department of Endocrinology and Metabolism and the Institute of Endocrinology, NHC Key Laboratory of Diagnosis and Treatment of Thyroid Diseases, First Hospital of China Medical University, Shenyang, China; 2 Department of Rheumatology and Immunology, First Hospital of China Medical University, Shenyang, China; 3 Department of Clinical Epidemiology and Evidence-based Medicine, First Hospital of China Medical University, Shenyang, China; 4 School of Public Health, Shenzhen University Medical School, Shenzhen, China

## Abstract

**Objectives:**

To estimate the burden, trends, and inequalities of type 1 diabetes mellitus (T1DM) among older adults at global, regional, and national level from 1990 to 2019.

**Design:**

Population based study.

**Population:**

Adults aged ≥65 years from 21 regions and 204 countries and territories (Global Burden of Disease and Risk Factors Study 2019)from 1990 to 2019.

**Main outcome measures:**

Primary outcomes were T1DM related age standardised prevalence, mortality, disability adjusted life years (DALYs), and average annual percentage change.

**Results:**

The global age standardised prevalence of T1DM among adults aged ≥65 years increased from 400 (95% uncertainty interval (UI) 332 to 476) per 100 000 population in 1990 to 514 (417 to 624) per 100 000 population in 2019, with an average annual trend of 0.86% (95% confidence interval (CI) 0.79% to 0.93%); while mortality decreased from 4.74 (95% UI 3.44 to 5.9) per 100 000 population to 3.54 (2.91 to 4.59) per 100 000 population, with an average annual trend of −1.00% (95% CI −1.09% to −0.91%), and age standardised DALYs decreased from 113 (95% UI 89 to 137) per 100 000 population to 103 (85 to 127) per 100 000 population, with an average annual trend of −0.33% (95% CI −0.41% to −0.25%). The most significant decrease in DALYs was observed among those aged <79 years: 65-69 (−0.44% per year (95% CI −0.53% to −0.34%)), 70-74 (−0.34% per year (−0.41% to −0.27%)), and 75-79 years (−0.42% per year (−0.58% to −0.26%)). Mortality fell 13 times faster in countries with a high sociodemographic index versus countries with a low-middle sociodemographic index (−2.17% per year (95% CI −2.31% to −2.02%) *v* −0.16% per year (−0.45% to 0.12%)). While the highest prevalence remained in high income North America, Australasia, and western Europe, the highest DALY rates were found in southern sub-Saharan Africa, Oceania, and the Caribbean. A high fasting plasma glucose level remained the highest risk factor for DALYs among older adults during 1990-2019.

**Conclusions:**

The life expectancy of older people with T1DM has increased since the 1990s along with a considerable decrease in associated mortality and DALYs. T1DM related mortality and DALYs were lower in women aged ≥65 years, those living in regions with a high sociodemographic index, and those aged <79 years. Management of high fasting plasma glucose remains a major challenge for older people with T1DM, and targeted clinical guidelines are needed.

## Introduction

Type 1 diabetes mellitus (T1DM) was traditionally considered a disease that manifested during childhood and adolescence and could have a profound effect on life span.^1^ ^2^ Variable evidence from recent studies suggests that the life expectancy of an increasing number of older and elderly people with T1DM has improved as has diabetes care and the management of complications.^3^ ^4^ Current and comprehensive data on the burden of T1DM is, however, lacking in most countries and territories worldwide.^5^ ^6^ Nearly all of the existing clinical practices and guidelines lack targeted content for the management of T1DM among older people.^7^

As the global population ages, substantial gaps in data related to T1DM need to be bridged urgently. A modelling study estimated that a diagnosis of T1DM was missed in 3.7 million people in 2021, and it predicted a 60-107% increase in the number of people with a diagnosis between 2021 and 2040 globally, highlighting the opportunity to raise the standard of care and save millions of lives in the coming decade.^8^ This increasing prevalence could represent an increasing number of people with T1DM living to elderly age. Importantly, based on current evidence, the number of older people with T1DM has already likely increased owing to an overall increase in life expectancy.^4^ Continuous T1DM care of people in older age, diabetes related complications, and impaired cognitive and physiological function from ageing are challenging for both people with T1DM and healthcare resources.^9^ Understanding the changes in mortality and DALYs among older people (≥65 years) with T1DM is critical for their care.

We investigated T1DM associated prevalence, mortality, and disability adjusted life years (DALYs) in adults aged ≥65 years at global, regional, and national level during 1990-2019 by social developmental level and age and sex of the older population. We also assessed factors that might have an influence on DALYs among older people with T1DM.

## Methods

### Study population and data collection

In our analysis of the Global Burden of Disease Study 2019, we accessed repeated cross sectional data from the Global Health Data Exchange, encompassing the global burden of 369 diseases and injuries and 87 risk factors, including T1DM, across 21 regions and 204 countries and territories from 1990 to 2019. The study population comprised people with T1DM aged 65 years and older, including those with diabetes diagnosed before age 65 years.^10^ ^11^ ^12^ ^13^ From the Global Burden of Disease Study 2019 we extracted information on T1DM in people aged ≥65 years; location, age, and sex specific prevalence; mortality; numbers and rates for DALYs; and DALYs attributable to each risk factor (with corresponding 95% uncertainty intervals (UIs)). Attributable DALYs are a metric for quantifying the contribution of specific risk factors to the burden of disease. DALYs signify the changes and reduction in current burden of disease if a change occurs to population level exposure to a particular risk factor. After adjusting for comorbidity, we used micro-simulation to obtain the final estimate of years lived with disability. Years of life lost were calculated by multiplying the estimated number of deaths from T1DM by the standard life expectancy at the age of death. DALYs were equal to the sum of years lived with disability and years of life lost. The methodology employed in the Global Burden of Disease Study 2019 is described elsewhere (also see supplementary file, methods section).^14^ ^15^

In the Global Burden of Disease Study, T1DM is defined as doctor diagnosed disease identified through a diabetic registry or hospital records. To estimate non-fatal burdens of T1DM and complications from T1DM, a bayesian meta-regression modelling tool, DisMod-MR 2.1, was used to analyse 1527 location years of data from the scientific literature, survey microdata, and insurance claims.^14^ Estimates of both non-fatal and fatal outcomes in people with T1DM include those attributed to complications from the disease. The Global Burden of Disease Study extrapolates and models data based on the reported T1DM, juvenile onset diabetes, and insulin dependent diabetes to develop a database applicable to most countries, with no age restrictions on the input raw data. To allow for smoothing over age, time, and location in areas without complete datasets, spatiotemporal Gaussian process regression was used to model the Global Burden of Disease Study 2019 input data.^14^ The Global Burden of Disease Study analysed the spatiotemporal Gaussian process regression.^16^ The methods section in the supplementary file provides detailed information on the input data and methodology for T1DM.

In this study, we collected data on T1DM from 21 regions of countries that are geographically proximate and have similar epidemiological profiles, encompassing seven age groups (65-69 years, 70-74 years, 75-79 years, 80-84 years, 85-89 years, 90-94 years, ≥95 years) for both men and women. We also calculated the sociodemographic index for each country, which is a composite indicator of the social and economic conditions influencing health outcomes in each locality. The sociodemographic index ranges from 0.005 to 1. In this study 1 represented the highest education level, highest per capita income, and lowest fertility rate. The sociodemographic index is divided into five categories: low, low-middle, middle, high-middle, and high.

### Statistical analysis

**Fig 1:**
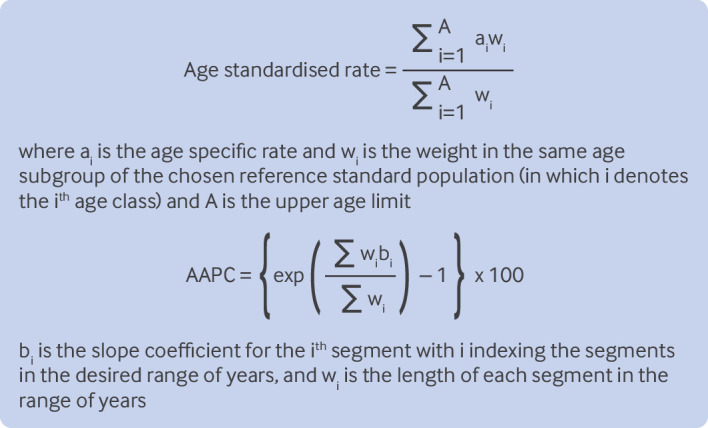
Equations used to calculate age standardised rate and average annual percentage change (AAPC)

A descriptive analysis was performed to characterise the burden of T1DM among adults aged ≥65 years on a global scale. We compared the age standardised prevalence (per 100 000 population), age standardised mortality (per 100 000 population), and age standardised DALYs (per 100 000 population) of T1DM across different age groups, sexes, regions, and countries. Based on the data on T1DM and associated risk factors obtained from the Global Burden of Disease Study, we further calculated the age standardised rates and corresponding 95% confidence intervals (CIs) based on the world standard population reported in the Global Burden of Disease Study 2017 for comparison between regions, and further estimated average annual percentage changes (AAPCs) by joinpoint regression to measure the temporal trend.^14^ Our estimates are shown per 100 000 population using the top equation in figure 1.

AAPCs are used to represent the average increase or rate of change of a specific variable over a specified period. In this study, it is the annual change percentage transformed from the weighted average of the slope coefficients of the underlying joinpoint regression model from 1990 to 2019.^17^ The AAPC value denotes the percentage annual change (increase, decrease, or no change). If annual percentage change estimates and 95% CIs were both >0 (or both <0), we considered the corresponding rate to be in an upward (or downward) trend. AAPC was calculated using the lower equation in figure 1.

The measures we chose are correlated epidemiologically, including prevalence, DALYs (one DALY represents the loss of one year of full health owing to premature death or disability), and mortality. For example, the reduction in mortality from a chronic disease could lead to an increase in both prevalence and DALYs, in terms of more patients living longer with that disease. An increased number of people with incident disease or a longer disease duration can both contribute to increased prevalence. The increasing prevalence of T1DM among people aged ≥65 years could be explained by longer life with disease because of improved medical care.

All statistical analyses were conducted using GraphPad Prism (version 8.0), Joinpoint Regression program (version 5.0.2), and R (version 4.2.3).

### Patient and public involvement

The Global Burden of Disease Study is a collaborative scientific effort allowing the effects of different health conditions to be compared and replicated between age, sex, and geographical locations for specific points in time. The study has generated substantial scientific, policy, and public interest worldwide. Our study used the secondary data from this collaborative work, and we did not have direct access to the participants. No patients were involved in setting the research question or the outcome measures, nor were they involved in the design and implementation of the study. *The BMJ* invited a patient reviewer as a member of the public to read our manuscript after submission.

## Results

### Global trends

Globally, the prevalence of T1DM among older people aged ≥65 years increased by 180% between 1990 and 2019, from 1.3 million to 3.7 million. The age standardised prevalence rate of T1DM among this age group increased by 28%, from 400 per 100 000 population in 1990 to 514 per 100 000 population in 2019, with an average annual trend of 0.86% (table 1). Furthermore, the proportion of older people with T1DM has shown a consistent upward trend relative to the overall number of people with T1DM, increasing from 12% in 1990 to 17% in 2019 (see supplementary figure 1). Compared with the overall number of people with T1DM, the increasing trend in prevalence of T1DM among those aged ≥65 years was noticeable from 1990 to 2019 (see supplementary figure 2). The disease burden (all cause DALYs) from T1DM has been increasing over the past 30 years in the population aged ≥65 years (see supplementary figure 3). The age standardised mortality from T1DM among this age group significantly decreased by 25%, from 4.7 per 100 000 population in 1990 to 3.5 per 100 000 population in 2019, with an average annual trend of −1.00% (see supplementary table 1).

**Table 1:** Age standardised prevalence and AAPC of T1DM in people aged ≥65 years at global and regional level, 1990-2019

	Prevalence (95% UI)				AAPC (95% CI))
	No of people with T1DM in 1990 (000s)	Age standardised rate in 1990 (per 100 000)	No of people with T1DM in 2019 (000s)	Age standardised rate in 2019 (per 100 000)	
Global	1284 (1064 to 1527)	400 (332 to 476)	3688 (2992 to 4478)	514 (417 to 624)	0.86 (0.79 to 0.93)
Sex:					
Female	734 (608 to 871)	399 (331 to 474)	1955 (1587 to 2370)	495 (402 to 600)	0.74 (0.65 to 0.83)
Male	550 (457 to 657)	401 (333 to 479)	1733 (1400 to 2111)	536 (433 to 653)	1.00 (0.95 to 1.06)
Age group (years):					
65-69	456 (377 to 543)	369 (305 to 440)	1218 (986 to 1490)	471 (381 to 575)	0.83 (0.72 to 0.93)
70-74	325 (269 to 386)	384 (318 to 457)	941 (765 to 1140)	503 (409 to 611)	0.94 (0.85 to 1.04)
75-79	254 (212 to 302)	414 (346 to 492)	668 (543 to 805)	526 (428 to 633)	0.82 (0.76 to 0.89)
80-84	153 (127 to 181)	434 (362 to 514)	464 (378 to 562)	550 (448 to 665)	0.82 (0.75 to 0.89)
85-89	69.1 (57.1 to 82.2)	459 (379 to 546)	257 (207 to 311)	590 (475 to 715)	0.86 (0.72 to 1.01)
90-94	21.8 (17.9 to 26.0)	495 (405 to 590)	108 (86.9 to 131)	642 (516 to 778)	0.89 (0.79 to 1.00)
≥95	5.32 (4.29 to 6.44)	517 (417 to 626)	31.9 (25.6 to 38.7)	668 (535 to 810)	0.88 (0.82 to 0.94)
SDI level:					
High	678 (570 to 794)	688 (578 to 805)	1666 (1361 to 2012)	913 (746 to 1103)	0.98 (0.93 to 1.03)
High-middle	327 (269 to 392)	369 (303 to 442)	956 (770 to 1167)	519 (418 to 634)	1.19 (1.09 to 1.28)
Middle	129 (103 to 158)	165 (132 to 203)	561 (450 to 692)	272 (218 to 335)	1.73 (1.66 to 1.80)
Low-middle	104 (82.5 to 128)	236 (186 to 293)	374 (297 to 461)	336 (266 to 415)	1.21 (1.12 to 1.29)
Low	45.9 (36.2 to 57.2)	263 (206 to 329)	130 (103 to 161)	338 (267 to 420)	0.86 (0.85 to 0.88)

AAPC=average annual percentage change; CI=confidence interval; SDI=sociodemographic index; T1DM=type 1 diabetes mellitus; UI=uncertainty interval.

Compared with prevalence and mortality, the trend of DALYs from T1DM among people aged ≥65 years was less noticeable during the same period. Age standardised DALYs decreased by 8.9%, from 113 per 100 000 population in 1990 to 103 per 100 000 population in 2019, with an average annual trend of −0.33% (see supplementary table 1).

### Global trends by sex

From 1990 to 2019, the age standardised prevalence of T1DM among people aged ≥65 years increased for both men and women worldwide (men: from 0.6 million to 1.7 million; women: from 0.7 million to 2 million). The increase in T1DM prevalence was more rapid among men than among women (AAPC 1.00% *v* 0.74%) (table 1). During the same period, the age standardised mortality from T1DM decreased for both men and women aged ≥65 years, although the reduction was smaller in men (AAPC −0.58% *v* −1.29%) (see supplementary table 1).

From 1990 to 2019, the reduction in age standardised DALYs from T1DM among older people was less pronounced in men than in women. The decrease in DALYs was more substantial in women aged ≥65 years, with an average annual trend of −0.58%. In 1990, among people aged ≥65 years, women had higher DALYs from T1DM per 100 000 population than men (118 *v* 106). In 2019, however, women aged ≥65 years had fewer DALYs from T1DM per 100 000 population than men (100 *v* 106) (see supplementary table 1). This sex difference remained consistent regardless of changes in sociodemographic index, with the burden of diseases higher in men compared with women, especially in countries with a low-middle sociodemographic index (see supplementary figures 4 and 5).

### Global trends by age subgroup

Globally, during 1990-2019 the prevalence of T1DM at least tripled in every age subgroup of people aged ≥65 years (65-69 years: from 0.46 million to 1.2 million; 70-74 years: from 0.32 million to 0.94 million; 75-79 years: from 0.25 million to 0.67 million; 80-84 years: from 0.15 million to 0.46 million; 85-89 years: from 0.07 million to 0.26 million) and even increased 5-6 times for those >90 years (90-94 years: from 0.02 million to 0.11 million; ≥95 years: from 0.005 million to 0.03 million). Notably, among all age subgroups, the upward trend for men consistently surpassed that for women, especially among men aged 90-94 years (AAPC 1.18%) (see supplementary figure 6). The age standardised prevalence of T1DM across all age subgroups increased at a noticeable rate during 1990-2019, especially among those aged 70-74 years (AAPC 0.94%) (table 1). The age standardised mortality of T1DM decreased across all age subgroups among older people worldwide, especially among those younger than 79 years. In 2019, the mortality rates for T1DM per 100 000 population among people aged ≥65 years increased with age, from 2.8 among those aged 65-69 years to 10 among those aged ≥95 years (see supplementary table 1).

The age standardised DALYs associated with T1DM among older people decreased in all age subgroups at various rates. The most significant decrease was observed among those aged <79 years, including 65-69 years (AAPC −0.44%), 70-74 years (−0.34%), and 75-79 years (−0.42%). In 2019, the DALYs attributable to T1DM were highest in those aged 75-79 years (111 per 100 000 population) and ≥95 years (111 per 100 000 population) (see supplementary table 1).

### Global trends by sociodemographic index

The age standardised prevalence of T1DM among people aged ≥65 years increased across all subgroups of sociodemographic index during 1990-2019, especially in countries with a middle sociodemographic index (AAPC 1.73%). Regardless of sociodemographic level, the increase in prevalence among those aged ≥65 years has consistently been higher than in the general population (see supplementary figures 7 and 8). In 2019, among older people the highest prevalence of T1DM was in countries with a high sociodemographic index (913 per 100 000 population) (table 1). While T1DM related mortality among older people decreased across all sociodemographic subgroups during the same period, the largest decrease was attributed to countries with a high sociodemographic index (AAPC −2.17%), which was more than 13 times faster than that in countries with a low-middle sociodemographic index (AAPC −0.16%). In 2019, the highest mortality rate was in countries with a low sociodemographic index (6.4 per 100 000 population), which was more than two times the mortality in countries with a high sociodemographic index (2.2 per 100 000 population) (see supplementary table 1 and supplementary figures 4 and 5).

The age standardised DALYs from T1DM among older people significantly decreased across all sociodemographic subgroups, except in countries with a low-middle sociodemographic index (AAPC 0.01%) (see supplementary table 1). The downward trend in DALYs for T1DM became more pronounced after surpassing a sociodemographic index threshold of 0.7 (see supplementary figure 9). In 2019, the DALYs were highest in countries with a low sociodemographic index (141 per 100 000 population) and lowest in countries with a high-middle sociodemographic index (86 per 100 000 population) (see supplementary table 1).

### Regional trends

From 1990 to 2019, none of the 21 regions showed a decrease in prevalence of T1DM among people aged ≥65 years. The most rapid increase in age standardised prevalence for older with T1DM was observed in North Africa and the Middle East (AAPC 2.40%), East Asia (2.17%), and western Europe (1.95%), and the slowest increase in prevalence was in high income North America (0.60%) during the same period (see supplementary figure 10). In 2019, among the 21 regions, the highest age standardised prevalence for T1DM among people aged ≥65 years was in high income North America (1248 per 100 000 population), Australasia (1080 per 100 000 population), and western Europe (1077 per 100 000 population) (see supplementary table 2). No difference was observed after sex stratification (see supplementary table 3).

Most regions experienced a reduction in DALYs from T1DM among older people at various rates during 1990-2019. However, a large increase in DALYs was found in central Asia (AAPC 1.79%). The largest reduction in DALYs from T1DM among older people was in Central Latin America (AAPC −1.30%) (see supplementary figure 10). In 2019, the highest DALYs from T1DM among older people were in southern sub-Saharan Africa (178 per 100 000 population), Oceania (178 per 100 000 population), and the Caribbean (177 per 100 000 population). The lowest DALYs were in East Asia (32 per 100 000 population), the high income Asia Pacific region (51 per 100 000 population), and eastern Europe (77 per 100 000 population) (see supplementary table 4). After stratifying by sex, no significant differences were found (see supplementary figure 11).

### National trends

**Fig 2:**
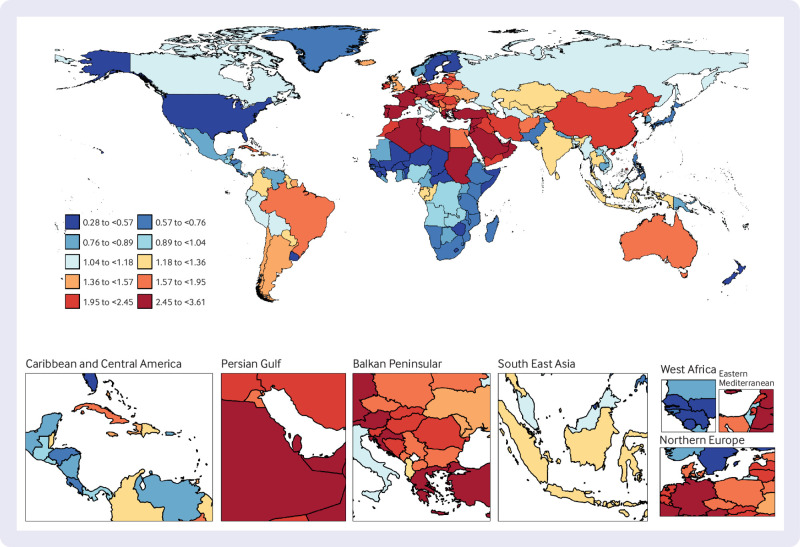
Map showing average annual percentage change in global prevalence of type 1 diabetes mellitus among people aged ≥65 years, 1990-2019

**Fig 3:**
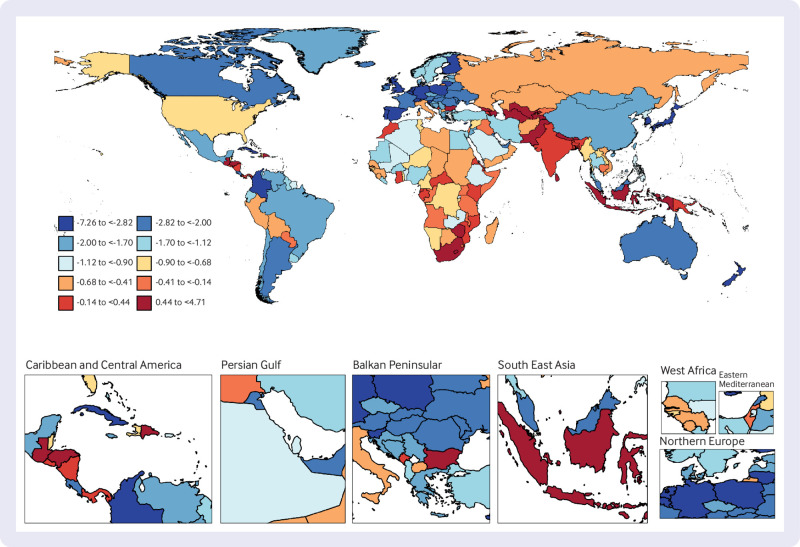
Map showing average annual percentage change in global mortality among people with type 1 diabetes mellitus aged ≥65 years, 1990-2019

**Fig 4:**
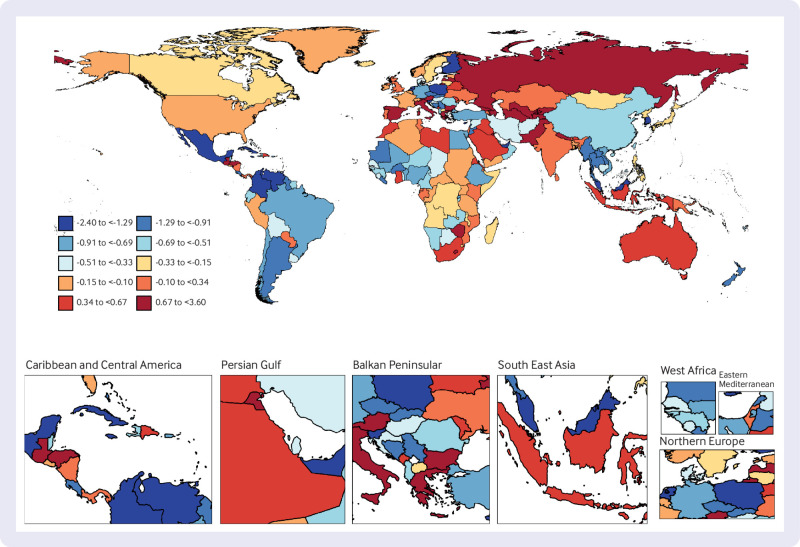
Map showing average annual percentage change in global DALYs among people with type 1 diabetes mellitus aged ≥65 years, 1990-2019

At the national level, from 1990 to 2019, United Arab Emirates had the highest increase in age standardised prevalence of T1DM among people aged ≥65 years, with an average annual trend of 3.61%, followed by Libya (AAPC 3.28%) and Greece (3.16%). Over the same period, Cuba showed the most substantial decrease in age standardised DALYs for older people with T1DM (AAPC −2.40%), followed by the Republic of Korea (−2.29%). The country with the greatest increase in age standardised DALYs for older people with T1DM was Uzbekistan (AAPC 3.60%). In 2019, Finland had the highest age standardised prevalence of T1DM among older people (1693 per 100 000 population), while Oman had the highest age standardised DALYs for T1DM among older people (418 per 100 000 population) (fig 2, fig 3, fig 4, and supplementary table 5).

### Risk factors

A detailed analysis of global data from 1990 to 2019 revealed three primary risk factors associated with DALYs for T1DM among people aged ≥65 years, including high fasting plasma glucose levels, low temperature, and high temperature. In 2019, these factors accounted for 103 per 100 000 people, 3 per 100 000 people, and 1 DALY per 100 000 people, respectively. From 1990 to 2019, the corresponding AAPCs for these factors were −0.33%, −2.27%, and 1.92%. In countries with a high sociodemographic index, the most substantial reduced burden from 1990 to 2019 was associated with a high fasting plasma glucose level (AAPC −0.37%) and low temperature (−2.65%). In contrast, countries with a low sociodemographic index contributed the most to the burden attributed to the three risk factors (table 2).

**Table 2:** Main risk factors for age standardised T1DM related DALYs, and AAPC, among people aged ≥65 years, 1990-2019

Risk factors by SDI	Age standardised DALYs (per 100 000) (95% UI)		AAPC (95% CI))
	1990	2019	
**High fasting plasma glucose**			
Global	113 (89 to 137)	103 (85 to 127)	−0.33 (−0.41 to −0.25)
High SDI	121 (93 to 151)	109 (82 to 143)	−0.37 (−0.48 to −0.26)
High-middle SDI	95 (78 to 117)	86 (68 to 109)	−0.37 (−0.47 to −0.28)
Middle SDI	105 (82 to 132)	91 (77 to 119)	−0.48 (−0.55 to −0.41)
Low-middle SDI	127 (73 to 176)	128 (95 to 166)	0.01 (−0.17 to 0.20)
Low SDI	153 (84 to 230)	141 (95 to 184)	−0.29 (−0.40 to −0.18)
**Low temperature**			
Global	5 (3 to 8)	3 (1 to 5)	−2.27 (−2.57 to −1.97)
High SDI	8 (4 to 12)	4 (2 to 6)	−2.65 (−3.18 to −2.10)
High-middle SDI	6 (3 to 10)	3 (2 to 5)	−2.37 (−2.70 to −2.04)
Middle SDI	4 (2 to 5)	2 (1 to 3)	−1.84 (−2.11 to −1.57)
Low-middle SDI	3 (0.2 to 6)	2 (−0.2 to 5)	−0.89 (−1.09 to −0.70)
Low SDI	3 (0.2 to 8)	3 (−0.4 to 7)	−0.70 (−2.14 to 0.76)
**High temperature**			
Global	0.7 (−2 to 3)	1 (0.2 to 3)	1.92 (1.54 to 2.29)
High SDI	0.1 (−0.1 to 0.3)	0.1 (0.004 to 0.2)	0.28 (−0.79 to 1.35)
High-middle SDI	0.2 (−0.5 to 0.9)	0.3 (0.03 to 0.9)	1.92 (1.43 to 2.42)
Middle SDI	0.9 (−3 to 5)	1 (0.2 to 4)	1.09 (0.62 to 1.55)
Low-middle SDI	2 (−7 to 10)	4 (0.6 to 9)	1.62 (1.26 to 1.97)
Low SDI	2 (−11 to 13)	4 (−0.5 to 10)	1.91 (1.58 to 2.24)

AAPC=average annual percentage change; CI=confidence interval; DALYs=disability adjusted life years; SDI=sociodemographic index; T1DM=type 1 diabetes mellitus; UI=uncertainty interval.

## Discussion

Worldwide during 1990-2019, the age standardised prevalence of T1DM increased among older people aged ≥65 years, concomitant with a substantial decrease in associated mortality. The results suggest that T1DM is no longer a contributory factor in decreased life expectancy owing to improvements in medical care over the three decades. DALYs from T1DM among older people decreased at a much slower rate, with inequality identified between countries with different sociodemographic levels. Optimal blood glucose control remains challenging among older people. Our study extended the current understanding of the increasing global burden of T1DM by focusing on trends in older people (≥65 years) with T1DM. Our study also advocates for urgent attention to coping strategies for ageing populations and older people with T1DM, rational allocation of health resources, and the provision of targeted guidelines. The findings are important for health practice and future research, and provide optimistic evidence for all people with T1DM, especially those with a diagnosis at a young age. 

### Age differences in burden of T1DM among older people

Our study found that in 2019, possibly owing to notable advances in medicine, mortality from T1DM significantly decreased and the life expectancy of affected people improved. These findings may be related to recent achievements in development goals aimed at improving accessibility and coverage of healthcare services, as well as progress in economic growth, reduced poverty, and social protection efforts.^18^ We found that the prevalence of T1DM substantially increased in every age subgroup among those aged ≥65 years. Populations worldwide are ageing, and caring for older people with T1DM involves both healthy ageing and management of T1DM. In recent years, with the increasing prevalence and accessibility of scientific technologies, an increasing number of older people are turning to technology to improve the management of their diabetes.^19^ The widespread adoption of insulin analogues and the improved utilisation of insulin pumps, along with improved education about diabetes and its management, in countries such as France and Sweden, have contributed to the improvement of the disease burden associated with T1DM.^20^ ^21^ ^22^ Although no cure exists for T1DM, the disease is manageable.^23^

### Sociodemographic differences in burden of older people with T1DM

The DALYs from T1DM among people aged ≥65 years decreased slower than mortality and unequally between countries. Firstly, against a background of increasing T1DM prevalence, a small decrease in DALYs is not necessarily harmful because of the huge improvement in survival for this population, and after accounting for more morbidity. Secondly, most of the prevalent cases of T1DM occurred among older people in high income regions and countries and the highest DALYs occurred in the least developed regions and countries. As the sociodemographic index decreased, the burden of T1DM among older people appeared to gradually increase. This aligns with previous studies, which identified limited access to insulin and testing equipment as crucial reasons why people with T1DM in countries with a low sociodemographic index do not receive timely management, which leads to low awareness, poor treatment, low control rates, and silent progression and deterioration among patients.^24^ Although this unequal distribution of T1DM associated DALYs between countries with different sociodemographic index reduced in the past three decades, it was still substantial in 2019.

### Sex difference in burden of T1DM among older people

In people aged ≥65 years, we observed a significantly increased overall disease burden from T1DM among men compared with women. T1DM is an autoimmune disease that results from activation of immune cells that destroy insulin producing pancreatic beta cells, and sex hormones are major contributors to this sex difference in the onset and progression of T1DM. Evidence suggests that the prevalence of T1DM is higher in girls during prepuberty, but high in men after puberty, findings that have also been observed in animal models.^25^ This sex inequality, however, is attributed not only to physiological differences between the sexes in terms of anatomy and metabolic processes but also to lifestyle, educational attainment, socioeconomic factors, and cultural factors.^26^ Studies have suggested that women exhibit a more proactive approach to healthcare utilisation and disease management, showing a higher awareness of their condition and greater adherence to treatment. These factors could mitigate the risk of diabetes in women and reduce the incidence of complications.^27^ Additionally, high risk factors such as smoking and lack of physical activity may, to some extent, account for this trend shift. These risk factors are also considered important prognostic factors for T1DM. Population based interventions aimed at reducing the presence of these risk factors are therefore crucial for managing and mitigating the disease burden of T1DM.^28^ Consequently, consideration of further sex specific treatment strategies is necessary to derive enhanced treatment benefits, and treatment plans can be more targeted.

### Risk factors in burden of T1DM among older people

A high fasting plasma glucose level was identified as the major contributor to DALYs from T1DM among people aged ≥65 years, indicating that blood glucose control is still suboptimal and a challenge among this population. On the one hand, most people with T1DM are unable to remain within normal glucose range for a large part of the day.^29^ The difficulty of optimal blood glucose control for people lies in the dynamic adjustment of insulin dose shots, which depends on factors such as daily nutrient intake, dietary patterns, insulin sensitivity factors, and daily activity. Difficulties, such as adjustment and calculation of insulin sensitivity factors according to dietary patterns, become even more challenging for older people owing to changes in metabolism and other factors associated with ageing. On the other hand, doctors might prefer to deal with hyperglycaemia rather than hypoglycaemia when caring for older people with diabetes, because complications from hyperglycaemia take longer to manifest than hypoglycaemia, the latter leading to immediate adverse outcomes such as unconsciousness, falls, brain damage, and cardiovascular events. Tighter control measures also increase stress for older patients at home and their caregivers. The objective should be to aim for normoglycaemia without putting people at risk of hypoglycaemia.^30^ We therefore suggest active, but not tight, control of blood glucose, especially in people aged ≥65 years with T1DM. The development of self-management skills, education about nutrition, and training are crucial for people with T1DM and their caregivers, all of which should be highlighted in guidelines for managing T1DM in older people. The latest advances in a new hybrid close loop insulin delivery system have been found to be safe and efficient for glucose instability among young people with T1DM,^31^ but evidence for its use in older people with T1DM is lacking.

Additionally, we observed that extreme temperatures were major contributors to the burden of T1DM among older people. A previous study suggested a positive correlation between high and low temperatures and diabetes incidence and mortality, particularly among older people.^32^ Temperature is one of the main environmental factors, and changes in temperature can influence insulin sensitivity.^33^ ^34^ Another explanation might be that temperature affects fasting plasma glucose levels through alterations in dietary and exercise patterns, heightened susceptibility to infections, activation of the sympathetic nervous system, and the triggering of catecholamine secretion, leading to faster onset of diabetes.^35^ Also, not only does high or low temperature result in physiological complications but it also affects people’s daily routines, activities, and dietary habits—all important factors in the wellbeing of people with T1DM. Notably, we found harm to be more noticeable during extreme temperatures in countries with a low sociodemographic index. This finding could be attributed to the management of T1DM, as extreme temperatures can impair insulin storage and reduce insulin efficacy.^36^ The capacity to adapt to variations in temperature is closely associated with the economic status of individual countries.^37^ Despite forecasts indicating that the global economic burden of diabetes in the adult population will escalate to $20tn (£16tn; €18tn) by 2030, the unmet demands for diabetes care in middle and low income countries persist.^38^ ^39^

### Strengths and limitations of this study

For older people with T1DM and their families worldwide, the decreasing mortality and DALYs associated with this disease is encouraging. For policy makers, health resource preparedness is needed for the growing number of people with both T1DM and ageing related problems, especially in countries with a middle and low sociodemographic index. In clinical practice, older people with T1DM are encouraged to take active control of their blood glucose levels, with the development of self-management skills and education about nutrition, and this should be reinforced in guidelines. Suitable training is also required for doctors and caregivers. For future research, intervention for glycaemic control that proved effective among adolescents and young adults with T1DM needs to be examined for older people too, with evidence based strategies. More innovative studies are needed in the area of healthy ageing in people with T1DM, and the cost effective delivery of treatment and care.

This study has several limitations. Firstly, the data were extrapolated from countries with existing epidemiological data. For our T1DM model, we used DisMod-MR 2.1 to generate estimates for older age groups based on age pattern. Interpreting the results in the real world requires caution. Secondly, despite using rigorous statistical methods in our study, variations in health information systems and reporting mechanisms across countries and regions, particularly in low and middle income countries and in areas experiencing conflict, could lead to incomplete data and bias, potentially affecting the accuracy of the results. Thirdly, the data on disease burden includes a time lag. Fourthly, we primarily relied on modelling processes for the estimates in this study, and the choice of models and parameter settings could have influenced the results. Finally, the diagnosis of T1DM in older people presents challenges,^40^ ^41^ and variations in healthcare systems, healthcare policies, and medical practices across countries may have an affect on disease burden. As a result, further high quality real world research is needed to validate the findings of this study.

### Conclusions

Mortality and DALYs among older people (≥65 years) with T1DM decreased considerably from 1990 to 2019. Both were lower in women, those living in countries or regions with a high sociodemographic index, and those younger than 79 years. Management of high fasting plasma glucose levels remains a major challenge for older people with T1DM, and targeted clinical guidelines are needed.

**What is already known on this topic**

Type 1 diabetes mellitus (T1DM) has been considered a disease to have a deleterious effect on life expectancy
Diabetes care has improved substantially since the 1990s, with an increasing number of older people with T1DM reported in studies
In most countries and regions worldwide, comprehensive and current data on the burden of T1DM are lacking

**What this study adds**

Using the Global Burden of Disease Study model, this study found that the age standardised prevalence of T1DM among people aged ≥65 years worldwide increased during 1990-2019 concomitant with a substantial decrease in associated mortality
A high fasting plasma glucose level was the major contributor to disability adjusted life years, indicating that hyperglycaemia management remained challenging for the older people with T1DM
Mortality fell 13 times faster in countries with a high sociodemographic index versus countries with a low-middle sociodemographic index (−2.17% per year *v* −0.16% per year)

## Data Availability

The data used for analyses are publicly available at https://ghdx.healthdata.org/gbd-results-tool. All data will be made available on request to the corresponding author. Proposals will be reviewed and approved by the sponsor, investigator, and collaborators based on scientific merit. After approval of a proposal, data will be shared through a secure online platform after the signing of a data access agreement.

## Web extra

Extra material supplied by authors

Supplementary information: Additional methodology, tables, and figures, and the STROBE statement

## Notes

Contributors: KJY, XY, and CYJ contributed equally to this work. YZL and JZ contributed equally to this work and are joint senior authors. YZL and JZ conceived and designed the study. SND, HS, BM, and TTL supervised the study. KJY, XY, and CYJ performed the statistical analysis. All authors contributed to the acquisition, analysis, or interpretation of data. KJY, YZL, and JZ drafted the manuscript. All authors revised the report and approved the final version before submission. YZL is the guarantor and attests that all listed authors meet authorship criteria and that no others meeting the criteria have been omitted.

Transparency: The lead authors (YZL and JZ) affirm that the manuscript is an honest, accurate, and transparent account of the study being reported; that no important aspects of the study have been omitted; and that any discrepancies from the study as planned (and, if relevant, registered) have been explained.

Dissemination to participants and related patient and public communities: Our work will be distributed to clinicians and the wider community by a plain language summary through social media platforms. We will also disseminate our findings through presentations at international forums and academic societies.

Provenance and peer review: Not commissioned; externally peer reviewed.

Publisher’s note: Published maps are provided without any warranty of any kind, either express or implied. BMJ remains neutral with regard to jurisdictional claims in published maps.
