# Supplementary material for: Global burden of type 1 diabetes in adults aged 65 years and older, 1990-2019: population based study
Source: BMJ. 2024 Jun 12;385:e078432. doi: 10.1136/bmj-2023-078432 (PMC11167563; doi:10.1136/bmj-2023-078432)
Supplement: Supplementary file 1 — Supplementary information: Additional methodology, tables, and figures, and the STROBE statement [file yank078432.ww.pdf]

Supplementary Materials

Table of contents

Supplementary Methods. 3

1. Overview 3

2. Data sources 3

3. Calculation methods for DALYs 3

3.1 YLLs 4

3.2 YLDs 4

4. Estimation of type 1 diabetes mellitus non-fatal burdens in GBD 2019 7

4.1 Flowchart 7

4.2 Case definition 8

4.3 Data seeking 8

4.4 Data inputs 9

4.5 Data processing 9

4.6 Modelling Strategy 10

5. Estimation of type 1 diabetes mellitus fatal burdens in GBD 2019 10

5.1 Flowchart 10

5.2 Input data 11

5.3 Modelling strategy 11

5.4 Covariate selection 12

6. Estimation of type 1 diabetes mellitus burdens attributable to risk factor in GBD 2019 13

7. Processing of missing data 14

8. Data accuracy 14

8.1 Data extractions in each country 14

Reference: 14

Additional Results in Tables and Figure 16

Supplementary Table 1. Age-standardized mortality and DALYs of type 1 diabetes mellitus in elderly people and their AAPCs from 1990 to 2019 at the global and SDI levels... **16**

Supplementary Table 2. Age-standardized prevalence of type 1 diabetes mellitus in elderly people and their AAPCs from 1990 to 2019 at regional levels. **18**

Supplementary Table 3. Age-standardized prevalence, mortality and DALYs of type 1 diabetes mellitus in elderly people in 2019 at regional levels by sex. **20**

Supplementary Table 4. Age-standardized mortality and DALYs of type 1 diabetes mellitus in elderly people and their AAPCs from 1990 to 2019 at the regional levels **22**

Supplementary Table 5. Age-standardized prevalence, mortality, and DALYs of type 1 diabetes mellitus in elderly people in 2019 and their AAPCs between 1990-2019 in 204 countries and territories **25**

Supplementary Figure 1. The changes in the proportion of prevalence cases among type 1 diabetes mellitus patients (T1DM) aged over 65 years to the overall T1DM patients from 1990 to 2019. .... **33**
Supplementary Figure 2. Temporal trend of age-standardized prevalence (A) mortality (B) and disability-adjusted life years (C) for type 1 diabetes mellitus patients aged over 65 years and overall T1DM patients from 1990 to 2019. .... **34**
Supplementary Figure 3. The age-standardized mortality (A) and disability-adjusted life years (B) proportion of type 1 diabetes mellitus to all-cause in patients aged over 65 years from 1990 to 2019. .... **35**
Supplementary Figure 4. Temporal trend of age-standardized mortality and disability-adjusted life years of type 1 diabetes mellitus in elderly people from 1990 to 2019 at global and socio-demographic index levels by sex.. **36**
Supplementary Figure 5. Average annual percent changes of age-standardized mortality and disability-adjusted life years of type 1 diabetes mellitus in elderly people aged over 65 years from 1990 to 2019 at socio-demographic index levels by sex. **37**
Supplementary Figure 6. Average annual percent changes of age-standardized prevalence, mortality and disability-adjusted life years of type 1 diabetes mellitus in elderly people from 1990 to 2019 by sex and age. **38**
Supplementary Figure 7. Temporal trend of age-standardized prevalence, mortality and disability-adjusted life years of type 1 diabetes mellitus aged over 65 years and overall T1DM patients from 1990 to 2019 at global and socio-demographic index levels......... **39**
Supplementary Figure 8. Average annual percent changes of age-standardized prevalence (A), mortality (B) and disability-adjusted life years (C) of type 1 diabetes mellitus aged over 65 years and overall T1DM patients from 1990 to 2019 at global and socio-demographic index levels... **40**
Supplementary Figure 9. Prevalence (A), mortality (B) and disability-adjusted life years (C) rate of type 1 diabetes mellitus in elderly people from 204 countries according to the socio-demographic index in 2019.. **41**
Supplementary Figure 10. Average annual percent changes of age-standardized prevalence, mortality and disability-adjusted life years of type 1 diabetes mellitus in elderly people aged over 65 years from 1990 to 2019 at regions levels.......................... **42**
Supplementary Figure 11. Average annual percent changes of age-standardized mortality and disability-adjusted life years of type 1 diabetes mellitus in elderly people aged over 65 years from 1990 to 2019 at regions levels by sex. **43**
STROBE Statement **44**

Supplementary Methods.

1. Overview

The Global Burden of Disease (GBD) is an approach to global descriptive epidemiology.[1] It is a systematic, scientific effort to quantify the comparative magnitude of health loss due to diseases, injuries, and risk factors by age, sex, and geography for specific points in time. Institute for Health Metrics and Evaluation (IHME) serves as the coordinating center for the GBD and affiliated projects. Published in The Lancet in October 2020, GBD 2019 provides, for the first time, an independent estimation of population for each of 204 countries and territories and for the globe using a standardized, replicable approach, as well as a comprehensive update on fertility and migration.[1] GBD 2019 incorporates major data additions and improvements and methodological refinements. Mortality and life expectancy estimates have expanded to a total of 990 locations at the most detailed level, and new causes have been added to the fatal and nonfatal cause lists, for a total of 369 diseases and injuries (http://www.healthdata.org/gbd/about/protocol). GBD 2019 estimated each epidemiological quantity of interest—incidence, prevalence, mortality, years lived with disability (YLDs), years of life lost (YLLs), and disability-adjusted life-years (DALYs)—for 23 age groups; males, females, and both sexes combined; and 204 countries and territories that were grouped into 21 regions and seven super-regions. The GBD 2019 location hierarchy now includes all WHO member states. The GBD disease and injury analytical framework generated estimates for every year from 1990 to 2019. Diseases and injuries were organized into a levelled cause hierarchy from the three broadest causes of death and disability at Level 1 to the most specific causes at Level 4. Within the three Level 1 causes—communicable, maternal, neonatal, and nutritional diseases; noncommunicable diseases; and injuries—there are 22 Level 2 causes, 174 Level 3 causes, and 301 Level 4 causes (including 131 Level 3 causes that are not further disaggregated at Level 4). In total, 364 causes are nonfatal and 286 are fatal.[1]

2. Data sources

GBD 2019 synthesises a large and growing number of data input sources including surveys, censuses, vital statistics, and other health-related data sources. The data from these sources are used to estimate morbidity; illness, and injury; and attributable risk for 204 countries and territories from 1990 to 2019; mortality deaths are estimated from 1980 to 2019.

The GBD estimation process is based on identifying multiple relevant data sources for each disease or injury, including censuses, household surveys, civil registration and vital statistics, disease registries, health service use, air pollution monitors, satellite imaging, disease notifications, and other sources. Each of these types of data is identified from a systematic review of published studies, searches of government and international organization websites, published reports, primary data sources such as the Demographic and Health Surveys, and contributions of datasets by GBD collaborators. All data used in this study were extracted from the Global Health Data Exchange (http://ghdx.healthdata.org/gbd-results-tool) including (1) global age- and sex- specific prevalence, mortality, DALYs numbers and crude rates (per 100,000 persons) from 1990 to 2019; (2) Regional age- and sex- specific incidence, mortality, DALYs numbers and crude rates from 1990 to 2019 by socio-demographic index (SDI) categories; (3) National age- and sex- specific incidence, mortality, DALYs numbers and crude rates from 1990 to 2019; (4) GBD world standard population in 2017; (5) Age- and sex- specific type 1 diabetes mellitus (T1DM) DALYs numbers and crude rates attributable to risk factors (level 4).

3. Calculation methods for DALYs

To estimate DALYs, GBD 2019 started by estimating cause-specific mortality and non-fatal health loss. For each year for which YLDs have been estimated, GBD 2019 computed

DALYs by adding YLLs and YLDs for each age-sex-location. Uncertainty in YLLs was assumed to be independent of uncertainty in YLDs. GBD 2019 calculated 1000 draws for DALYs by summing the first draw of the 1000 draws for YLLs and YLDs and then repeating for each subsequent draw. 95% UIs were computed by using the 25th and 975th ordered draw of the DALY uncertainty distribution. GBD 2019 calculated DALYs as the sum of YLLs and YLDs for each cause, location, age group, sex, and year. For more information, please refer to the following figure A.

Figure A. DALY burden estimation for GBD 2019

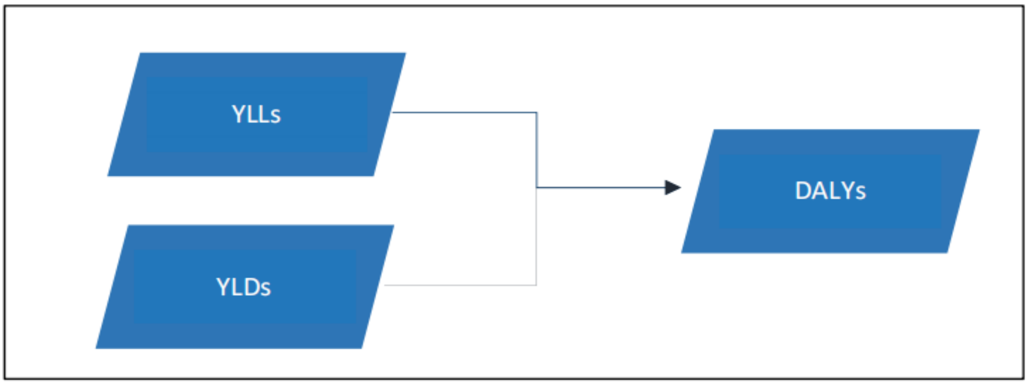

3.1 YLLs

The YLL is a metric that is computed by multiplying the number of estimated deaths by the standard life expectancy at age of death. The metric therefore highlights premature deaths by applying a larger weight to deaths that occur in younger age groups. The core equation can be written as follows:

$$YLL = \sum_{c=1,a=0,s=1}^{\infty} d_{cas}e_a$$

3.2 YLDs

YLDs was computed by sequela as prevalence multiplied by the DW for the health state associated with that sequela.

$$YLD\ Rate_k = \frac{\sum_{l=1}^{n} ADW_{LK}}{n}$$

3.2.1 Disability Weights (DWs)

DWs are measured on a scale from 0 to 1; 0 implies a state equivalent to full health, and 1, a state equivalent to death.

The formula for the cumulative DW is one minus the multiplicative sum of one minus each DW present:

$$Simulant\ DW_l = 1 - \prod_{k=i}^{j}(1 - DW_k)$$

Where:

DW_k is the DW for the kth disease sequela that the simulant l has acquired.

Once the simulant DW is computed, the DW attributable to each sequela for the simulant is calculated by using the following formula:

$$ADW_{lk} = \frac{DW_k}{\sum_{k=i}^{k=j} DW_k} * Simulant\ DW_l$$

Where:
ADW_{lk} is the attributable DW for disease sequela *k* in simulant *l*
DW_k is the DW for disease sequela *k*
Simulant DW_l is the DW for simulant *l* from the combination of all sequelae that they have acquired.
This formula apportions the overall simulant DW to each condition in proportion to the DW of each condition in isolation.

Finally, YLDs per capita in an age-sex-country-year are computed by taking the sum of the attributable DWs for a disease sequela across simulants.
The actual number of YLDs from disease sequela *k* in an age-sex-location-year is then computed as the YLD rate *k* times the appropriate age-sex-location-year population.

GBD 2019 determined the disability weights for each sequela from the GBD disability weight survey. The table below illustrates the severity levels, lay descriptions, and associated disability weights applicable for outcomes related to T1DM and T2DM.

Severity level	Lay description	DW (95% CI)
Uncomplicated Diabetes Mellitus	Has a chronic disease that requires medication every day and causes some worry, but minimal interference with daily activities	0.049 (0.031 – 0.072)
Diabetic neuropathy	Has pain, tingling, and numbness in the arms, legs, hands, and feet. The person sometimes gets cramps and muscle weakness.	0.133 (0.089 – 0.187)
Diabetic neuropathy with diabetic foot	Has a sore on the foot that is swollen and causes some difficulty in walking.	[a]
Diabetic neuropathy with treated amputation	Has lost part of one leg, leaving pain and tingling in the stump. The person has an artificial leg that helps in moving around.	[a]
Diabetic neuropathy with untreated amputation	Has lost part of one leg, leaving pain and tingling in the stump. The person does not have an artificial leg, has frequent sores, and uses crutches.	[a]
Moderate vision loss due to Diabetes Mellitus	Has vision problems that make it difficult to recognize faces or objects across a room.	0.031 (0.019 – 0.049)
Severe vision loss due to Diabetes Mellitus	Has severe vision loss, which causes difficulty in daily activities, some emotional impact (for example worry), and some difficulty going outside the home without assistance.	0.184 (0.125 – 0.259)
Blindness due to Diabetes Mellitus	Is completely blind, which causes great difficulty in some daily activities, worry and anxiety, and great difficulty going outside the home without assistance.	0.187 (0.124 – 0.26)

a: The disability weights are produced from a combination of two health states: neuropathy and diabetic foot/amputation.

4. Estimation of type 1 diabetes mellitus non-fatal burdens in GBD 2019

4.1 Flowchart

Calculating prevalence of type 1 diabetes mellitus

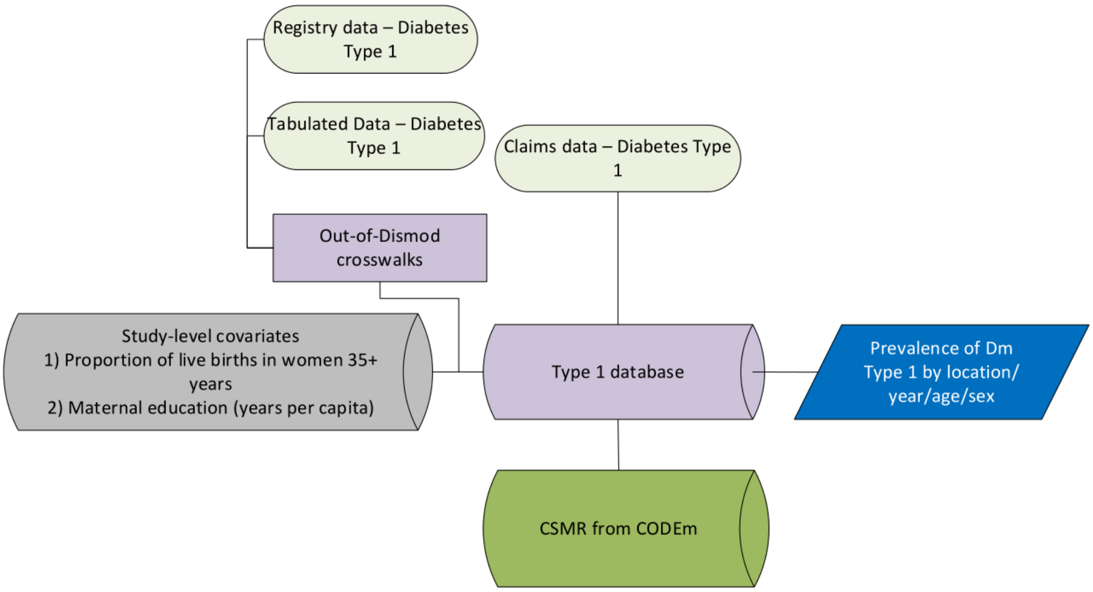

4.2 Case definition

Diabetes Mellitus Type 1

Criterion	Definition
1. Diabetes Mellitus Type 1	Cases of type 1 DM diagnosed by physicians and identified through a diabetic registry or hospital records
2. Uncomplicated Diabetes Mellitus Type 1	Cases of type 1 DM that do not have any of the following complications: neuropathy, foot ulcer, leg amputation, or vision loss
3. Diabetic neuropathy among Diabetes Mellitus Type 1	Cases of type 1 DM that experience diagnosable neuropathy
4. Diabetic foot due to neuropathy among Diabetes Mellitus Type 1	Cases of type 1 DM that currently have a foot ulcer
5. Diabetic neuropathy and amputation with treatment among Diabetes Mellitus Type 1	Cases of type 1 DM that have had a leg amputation above or below the knee, with treatment consisting of a prosthetic limb
6. Diabetic neuropathy and amputation without treatment among Diabetes Mellitus Type 1	Cases of type 1 DM that have had a leg amputation above or below the knee, with no prosthetic limb
7. Moderate vision impairment due to Diabetes Mellitus Type 1	Cases of type 1 DM that have moderate vision loss due to diabetic retinopathy
8. Severe vision impairment due to Diabetes Mellitus Type 1	Cases of type 1 DM that have severe vision loss due to diabetic retinopathy
9. Blindness due to Diabetes Mellitus Type 1	Cases of type 1 DM that have blindness due to diabetic retinopathy

4.3 Data seeking

First, a systematic review of the literature was done for GBD 2019. Second, GBD 2019 systematically searched the Global Health Data Exchange (GHDx) for multi-country survey programs, national surveys, and longitudinal studies that were tagged with either fasting plasma glucose (FPG) or Diabetes Mellitus. Finally, to capture any remaining sources not identified in the GHDx or in PubMed, they looked to other leaders in the field to ensure our datasets were as comprehensive as possible. These included data sources used by other research groups that report on the global burden of diabetes[2,3], microdata from not-yet published national studies, and publications that were not captured in the PubMed searchstring.

Figure 1: PRISMA diagram of data sources used in GBD 2019 Diabetes Mellitus model

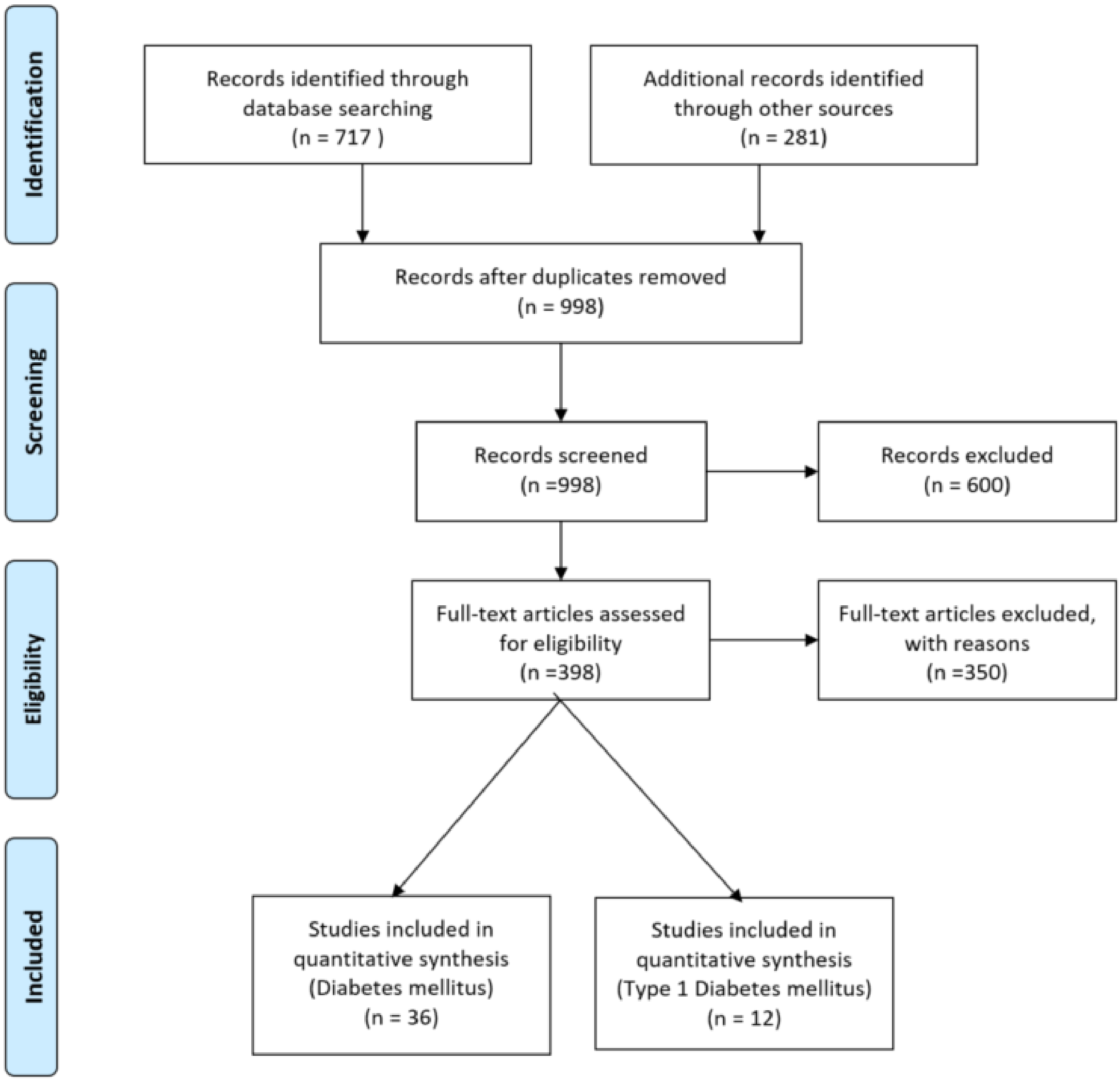

4.4 Data inputs

Purpose:
To incorporate all available data related to population-representative estimates of T1DM, GBD 2019 accepted data that reported T1DM, juvenile-onset Diabetes, and insulin-dependent Diabetes.
Data:
Data inputs comes from 2 types of sources:
• Estimates of T1DM in a representative population
• Diabetic registries

4.5 Data processing

Based on assumption that claims data in persons <15 years are T1DM and that 100% of

diabetics are captured in this age group, GBD 2019 make no adjustments to data in these ages. Claims data are reported as prevalence.

There are a number of different sources and ascertainment methods that were used to identify type 1 diabetics. The majority of data that are reported in the literature are from a diabetic registry, hospital discharge data review, physician interview, or insulin use. GBD 2019 assumed that there is no systematic bias between these sources and consider sources identified through these methods as reference. For the other sources that use alternative ascertainment techniques (eg., pharmacy reports, diabetic camps, school reports), there was not sufficient amount of data to perform an analysis on each individual type, and the model had relatively few data points in locations where these approaches were used. So they collapsed all alternative sources and treated the estimates from these sources as defined as an alternative case definition.

Table: Adjustment factors for alternative case definitions, from MR-BRT analysis

Alternative case definition	Beta coefficient	Lower	Upper
Ascertainment through pharmacy, schools, diabetic camps	0.9	0.80	1.10

4.6 Modelling Strategy

For GBD 2019, they estimated the overall prevalence of diabetes using DisMod MR-2.1, a Bayesian metaregression. They used data that reported incidence, standardized mortality ratio, and prevalence data in claims data for persons <15 years for T1DM. They decided to not include reported T1DM prevalence in non-claims sources because they found that their estimates of prevalence and incidence were inconsistent. They decided to trust the incidence data and thus, had to exclude the prevalence data from the model. Similarly, they did not include prevalence of T1DM in people >15 years from claims sources, because of poor reporting on type of diabetes.

Model parameters and estimates

• They set a value prior of 0 for remission for all ages

Country covariate	Parameter	beta	Exponentiated beta
Proportion of live births in women 35+ years	Incidence	2.60 (2.34-2.88)	13.42 (10.34 – 17.78)
Maternal education (years per capita)	Incidence	0.091 (0.083 – 0.10)	1.10 (1.09 – 1.11)

5. Estimation of type 1 diabetes mellitus fatal burdens in GBD 2019

5.1 Flowchart

Type 1 diabetes mellitus (T1DM)

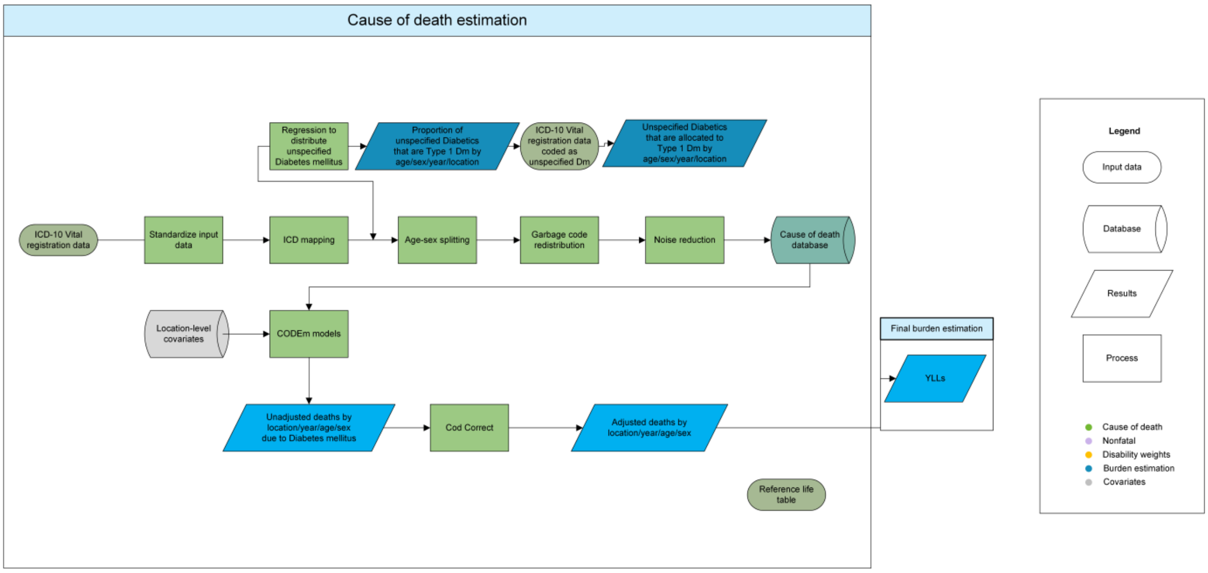

5.2 Input data

Type-specific diabetes mellitus mortality was estimated using deaths from vital registration sources in ICD-10 codes only. Diabetes type-specific information was not available in ICD-9 codes or deaths determined by verbal autopsy.

To incorporate all available data sources to estimate nonfatal burdens of diabetes, data that reported diabetes diagnosed by other measures of blood glucose (glycated hemoglobin A1c, oral glucose tolerance test, post prandial glucose test) were also included for the estimation of overall diabetes. For T1DM, data that reported T1DM, juvenile-onset diabetes, and insulin-dependent diabetes were all included. Considering the case definitions were not totally consistent between data sources, adjustment was applied in the modelling procedure. For example, majority of the data sources on T1DM in the GBD 2019 were identified through diabetic registry, hospital discharge data review, physician interview, or insulin use, which was considered as the reference method. For the other data sources that used alternative ascertainment techniques (e.g., pharmacy reports, diabetic camps, school reports), they were collapsed and the estimates were adjusted to the reference method in the modeling procedure.

5.3 Modelling strategy

The Cause of Death Ensemble model (CODEm) was used for deaths due to diabetes mellitus estimation.

Deaths in younger age groups are almost exclusively due to T1DM, while deaths in older ages are primarily due to type 2 diabetes mellitus (T2DM). To account for this age pattern, GBD 2019 set the age range of the T1DM model to 0-95+ years and the age range of the T2DM model to 15-95+ years. They used the same covariates in the T1DM model and T2DM model as the 0-14 year and 15-95+ year in the overall diabetes models, respectively.

There were two unique data manipulation steps that occurred in order to prepare the data as part of the modelling process.

1. GBD 2019 assumed that all deaths <15 years were due to type 1 regardless of the ICD-10 code assigned to the death. They imposed 100% attribution of diabetes mellitus deaths in <15

years to T1DM.
2. ICD-10 diabetes data were reported as type 1, type 2, or unspecified. GBD 2019 developed a regression to estimate the fraction of unspecified diabetes mellitus that was type 1 and type 2. They only used data from 703 country-years to inform the regression. This is because these country-years had more than 50% of the deaths typed to type 1 or type 2 AND nearly 30% of type-specific deaths in people >25 years were coded to type 1. Since there was a separate regression to estimate the proportion of T1DM and T2DM, they scaled the predicted proportions to one. These scaled proportions were then applied to number of deaths coded to unspecified diabetes in each location, year, sex where ICD-10 data was reported.

Regression equation

Type 1:

$$logit\left(\frac{number\ type\ 1\ DM}{number\ total\ DM}\right) \sim logit\left(\frac{number\ unspecified\ DM}{number\ total\ DM}\right) + \beta_1 age\ group + \beta_2 age\text{-}st\ prev\ obesity * age\ group + age\text{-}st\ prev\ obesity$$

Type 2:

$$logit\left(\frac{number\ type\ 2\ DM}{number\ total\ DM}\right) \sim logit\left(\frac{number\ unspecified\ DM}{number\ total\ DM}\right) + \beta_1 age\ group + \beta_2 age\text{-}st\ prev\ obesity * age\ group + age\text{-}st\ prev\ obesity$$

5.4 Covariate selection

The following are the covariates included in the model. GBD 2019 selected the same covariates for the T1DM model as the 0-14 year diabetes model and the T2DM model as the 15-95+ year diabetes model. In GBD 2019, they made 2 updates. First, they changed 4 covariates to reflect the most current covariate available, proportion underweight to age-standardised underweight (weight-for-age) summary exposure variable, proportion stunting to age-standardised stunting (height-for-age) summary exposure variable, energy-adjusted grams of fruits to age- and sex-specific summary exposure variable for low fruit, and energy-adjusted grams of vegetables to age- and sex-specific summary exposure variable for low vegetables. Second, they selected a direction on covariates that they did not set a direction in previous GBD. They determined the direction based on the strength of the evidence.

Model	Level	Covariate	Direction
Type 1	1	Healthcare access and quality index	-
	3	Education years per capita	-
	2	Age-standardised fertility rate	+
	2	Latitude	+
	2	Age-standardised underweight (weight-for-age) summary exposure variable	-
	2	Percentage of births occurring in women >35 years old	+
	2	Percentage of births occurring in women >40 years old	+
	3	Socio-demographic Index	-
	2	Age-standardised stunting (height-for-age) summary exposure variable	-
	2	Mean birth weight	-
Type 2	1	Age-standardised mean fasting plasma glucose (mmol/L)	+
	1	Age-standardised prevalence of diabetes	+
	3	Education years per capita	-
	3	Lag-distributed income per capita	+
	1	Mean BMI	+
	2	Mean cholesterol	+
	2	Mean systolic blood pressure	+
	1	Prevalence of obesity	+
	2	Age- and sex-specific summary exposure variable for low fruit	-
	2	Energy-adjusted grams of sugar	+
	2	Age- and sex-specific summary exposure variable for low vegetables	-
	3	Healthcare access and quality index	-
	2	Age- and sex-specific summary exposure variable for alcohol use	+

6. Estimation of type 1 diabetes mellitus burdens attributable to risk factor in GBD 2019

Four key components are included in the estimation of the burden attributable to a given risk factor: the metric of burden being assessed (the number of deaths, YLLs, YLDs, or DALYs [the sum of YLLs and YLDs]); the exposure levels for a risk factor; the RR of a given outcome due to exposure; and the counterfactual level of risk factor exposure. Estimates of attributable burden as DALYs for risk-outcome pairs were generated by using the following model:

$$AB_{jasgt} = \sum_{o=1}^{w} DALY_{joasgt} PAF_{joasgt}$$

where AB_{jasgt} is the attributable burden for risk factor *j* for age group *a*, sex *s*, location *g*, and year *t*; $DALY_{jasgt}$ is total DALYs for cause *o* (of *w* relevant outcomes for risk factor *j*) for age group *a*, sex *s*, location *g*, and year *t*; and PAF_{jasgt} is the PAF for cause *o* due to risk factor *j* for age group *a*, sex *s*, location *g*, and year *t*. The proportions of deaths, YLLs, or YLDs attributable to a given risk factor or risk factor cluster were analogously computed by sequentially substituting each metric in place of DALYs in the equation provided.

Definitions in GBD2019 of high fasting plasma glucose, high temperature and low temperature were listed below:

1) High fasting plasma glucose was defined as serum fasting plasma glucose of greater than 4.8–5.4 mmol/L. This was calculated by taking the person-year weighted average of the

levels of FPG that were associated with the lowest risk of mortality in the pooled analyses of prospective cohort studies.

2) High temperature (heat) exposure was defined as exposure to temperatures warmer than the theoretical minimum risk exposure level (TMREL) and low temperature (cold) was defined as temperatures colder than this TMREL. The population-weighted mean TMREL is 25.6℃, with a range of 21.3–26.6℃.

7. Processing of missing data

The GBD 2019 input data were modelled by using Spatiotemporal Gaussian process regression (ST-GPR) modelling to allow for smoothing over age, time, and location in locations that were missing complete datasets. The flowchart showing the analytic steps can be found elsewhere.[4] The approach is a stochastic modelling technique that is designed to detect signals amidst noisy data. It also serves as a powerful tool for interpolating non-linear trends.[5-6] The Bayesian noise reduction algorithm was used to deal with zero counts and small number issues for rare causes.

8. Data accuracy

Since most countries globally lack sufficient data on elderly T1DM, especially in low-resource regions and countries, resulting in a data gap for firsthand epidemiological investigations. GBD 2019 study used the following steps to make the estimates as accurate as possible: (1) compiling data sources through data identification and extraction; (2) data adjustment; (3) estimation of prevalence and incidence by cause and sequelae by using DisMod-MR 2.1 or alternative modelling strategies for selected cause groups; (4) estimation by impairment; (5) severity distributions; (6) incorporation of disability weights (DWs); (7) comorbidity adjustment; and (8) the estimation of YLDs by sequelae and causes.

8.1 Data extractions in each country

Diabetes registries or hospital documentation are collected from hospitals, governments, surveys, and other databases around the real world. GBD extracts data from each country through the following steps: first, GBD Collaborators start by gathering health data from hospitals, governments, surveys, systematic review of the literature and other databases around the world. Second, the research teams clean and sort the data and use the disease model-Bayesian meta-regression (DisMod-MR 2.1) modeling tools to generate estimates for locations and years where data are not available. Finally, to capture any remaining sources not identified in the Global Health Data Exchange (GHDx) or in PubMed, they looked to other leaders in the field to ensure the datasets were as comprehensive as possible. There are over 11,000 Collaborators in the Network, located in more than 162 countries. GBD Collaborators come from a variety of different work sectors and institutions, including research and scientific institutions, to healthcare delivery and policy as well as multilateral organizations.

GBD generated elderly T1DM data using the DisMod-MR2.1 model. However, there is no age restrictions on the original dataset input to the model, and GBD derives estimates through steps such as cleaning, standardization, adjustment, model building, and calibration validation of the collected raw data.

Reference:

1. GBD 2019 Diseases and Injuries Collaborators. Global burden of 369 diseases and

injuries in 204 countries and territories, 1990-2019: a systematic analysis for the Global Burden of Disease Study 2019. Lancet 2020; 396:1204-1222.
2. Global, regional, and national comparative risk assessment of 84 behavioural, environmental and occupational, and metabolic risks or clusters of risks for 195 countries and territories, 1990–2017: a systematic analysis for the Global Burden of Disease Study 2017. The Lancet 2018; 392: 1923–94.
3. Global, regional, and national disability-adjusted life-years (DALYs) for 359 diseases and injuries and healthy life expectancy (HALE) for 195 countries and territories, 1990–2017: a systematic analysis for the Global Burden of Disease Study 2017. Lancet 2018; 392: 1859–922.
4. GBD 2019 Risk Factors Collaborators. Global burden of 87 risk factors in 204 countries and territories, 1990–2019: a systematic analysis for the Global Burden of Disease Study 2019. The Lancet.
5. Vasudevan S, Ramos F, Nettleton E, Durrant-Whyte H, Blair A. Gaussian Process modeling of large scale terrain. In: 2009 IEEE International Conference on Robotics and Automation. 2009: 1047–53.
6. Rasmussen CE, Williams CKI. Gaussian Processes for Machine Learning. Cambridge, Mass: The MIT Press, 2005.

Additional Results in Tables and Figure

Supplementary Table 1. Age-standardized mortality and DALYs of type 1 diabetes mellitus in elderly people and their AAPCs from 1990 to 2019 at the global and SDI levels.

	Mortality						DALYs					
	**Cases in 1990 (thousands)**	**Age-standardized rate in 1990 (per 100,000)**	**Cases in 2019 (thousands)**	**Age-standardized rate in 2019 (per 100,000)**	**AAPCs in rate, 1990-2019 (%/year)**	**P value**	**Cases in 1990 (thousands)**	**Age-standardized rate in 1990 (per 100,000)**	**Cases in 2019 (thousands)**	**Age-standardized rate in 2019 (per 100,000)**	**AAPCs in rate, 1990-2019 (%/year)**	**P value**
Global	14.9 (10.9 to 18.5)	4.74 (3.44 to 5.90)	25.1 (20.7 to 32.5)	3.54 (2.91 to 4.59)	-1.00 (-1.09 to -0.91)	<0.001	372 (292 to 452)	113 (89 to 137)	744 (614 to 918)	103 (85 to 127)	-0.33 (-0.41 to -0.25)	<0.001
Sex												
Females	9.22 (5.77 to 11.9)	5.08 (3.17 to 6.56)	13.8 (10.4 to 18.8)	3.50 (2.63 to 4.77)	-1.29 (-1.36 to -1.22)	<0.001	221 (160 to 273)	118 (85 to 146)	395 (316 to 495)	100 (80 to 125)	-0.58 (-0.67 to -0.49)	<0.001
Males	5.65 (4.13 to 8.07)	4.24 (3.07 to 6.03)	11.3 (9.25 to 16.2)	3.59 (2.91 to 5.11)	-0.58 (-0.67 to -0.48)	<0.001	151 (118 to 198)	106 (83 to 139)	349 (281 to 453)	106 (85 to 137)	0.002 (-0.05 to 0.06)	0.96
Age group												
65-69	4.75 (3.56 to 5.97)	3.85 (2.88 to 4.84)	7.33 (6.12 to 9.59)	2.83 (2.37 to 3.71)	-1.06 (-1.18 to -0.94)	<0.001	150 (117 to 183)	121 (95 to 148)	277 (231 to 341)	107 (89 to 132)	-0.44 (-0.53 to -0.34)	<0.001
70-74	3.59 (2.67 to 4.55)	4.25 (3.16 to 5.38)	5.81 (4.88 to 7.51)	3.10 (2.61 to 4.02)	-1.07 (-1.20 to -0.94)	<0.001	96.3 (76.0 to 116.2)	114 (90 to 138)	193 (159 to 239)	103 (85 to 127)	-0.34 (-0.41 to -0.27)	<0.001
75-79	3.67 (2.68	5.98 (4.36 to	5.50 (4.51	4.33 (3.55 to	-1.11 (-	<0.00	77.1 (59.9	126 (98 to	141 (117 to	111 (92 to	-0.42 (-	<0.00

	to 4.52)	7.37)	to 7.06)	5.56)	1.23 to -0.99)	1	to 92.6)	151)	172)	135)	0.58 to -0.26)	1
80-84	1.43 (1.03 to 1.77)	4.07 (2.94 to 5.02)	2.74 (2.28 to 3.59)	3.25 (2.70 to 4.25)	-0.77 (-0.92 to -0.62)	<0.001	29.6 (23.5 to 36.2)	84 (67 to 103)	71.5 (57.2 to 88.8)	85 (68 to 105)	0.03 (-0.04 to 0.11)	0.39
85-89	0.890 (0.609 to 1.07)	5.90 (4.04 to 7.12)	2.06 (1.64 to 2.63)	4.74 (3.77 to 6.04)	-0.74 (-0.92 to -0.56)	<0.001	13.8 (10.8 to 16.7)	92 (71 to 111)	40.1 (31.9 to 50.1)	92 (74 to 115)	0.04 (-0.13 to 0.21)	0.68
90-94	0.394 (0.257 to 0.476)	8.95 (5.82 to 10.8)	1.17 (0.881 to 1.50)	6.91 (5.22 to 8.89)	-0.87 (-0.97 to -0.76)	<0.001	4.59 (3.53 to 5.68)	104 (80 to 129)	17.2 (13.6 to 21.6)	102 (81 to 128)	-0.07 (-0.14 to 0.005)	0.07
95+	0.142 (0.0890 to 0.176)	13.81 (8.64 to 17.12)	0.501 (0.347 to 0.664)	10.5 (7.27 to 13.9)	-0.93 (-1.15 to -0.72)	<0.001	1.21 (0.905 to 1.50)	118 (88 to 145)	5.28 (4.13 to 6.66)	111 (87 to 139)	-0.21 (-0.29 to -0.12)	<0.001
SDI quintiles												
High SDI	4.01 (2.56 to 5.31)	4.07 (2.59 to 5.39)	4.09 (2.91 to 5.95)	2.17 (1.55 to 3.15)	-2.17 (-2.31 to -2.02)	<0.001	1207 (91.87 to 150)	121 (93 to 151)	198 (149 to 261)	109 (82 to 143)	-0.37 (-0.48 to -0.26)	<0.001
High-middle SDI	3.22 (2.49 to 4.18)	3.69 (2.83 to 4.79)	4.43 (3.64 to 5.98)	2.46 (2.01 to 3.31)	-1.38 (-1.56 to -1.20)	<0.001	86.7 (70.7 to 107)	95 (78 to 117)	159 (125 to 201)	86 (68 to 109)	-0.37 (-0.47 to -0.28)	<0.001
Middle SDI	3.89 (2.89 to 5.09)	5.65 (4.13 to 7.41)	8.03 (6.67 to 11.3)	4.19 (3.45 to 5.85)	-1.00 (-1.04 to -0.97)	<0.001	81.6 (63.7 to 102)	105 (82 to 132)	189 (159 to 248)	91 (77 to 119)	-0.48 (-0.55 to -0.41)	<0.001
Low-middle SDI	2.53 (1.26 to 3.70)	6.31 (3.16 to 9.23)	6.19 (4.15 to 8.46)	5.96 (3.98 to 8.14)	-0.16 (-0.45 to 0.12)	0.27	56.3 (32.4 to 78.2)	127 (73 to 176)	143 (106 to 185)	128 (95 to 166)	0.01 (-0.17 to 0.20)	0.89
Low SDI	1.20 (0.559 to 1.92)	7.40 (3.41 to 11.82)	2.33 (1.37 to 3.18)	6.43 (3.76 to 8.79)	-0.48 (-0.61 to -0.36)	<0.001	27.4 (15.0 to 41.0)	153 (84 to 230)	55.2 (37.2 to 71.9)	141 (95 to 184)	-0.29 (-0.40 to -0.18)	<0.001

Note: Estimates are for individuals aged over 65 years. AAPCs=average annual percent changes. CI=confidence interval. DALYs=disability-adjusted life years. P=P value for the significant test of AAPCs. SDI=Socio-demographic Index. Numbers in parentheses are 95% uncertainty intervals (Cases and age-standardized rate) and 95% confidence interval (AAPCs).

Supplementary Table 2. Age-standardized prevalence of type 1 diabetes mellitus in elderly people and their AAPCs from 1990 to 2019 at regional levels.

Regions	Prevalence					
	Cases in 1990 (thousands)	Age-standardized rate in 1990 (per 100,000)	Cases in 2019 (thousands)	Age-standardized rate in 2019 (per 100,000)	AAPCs in rate, 1990-2019 (%/year)	P value
Andean Latin America	3.50 (2.79 to 4.31)	217 (173 to 268)	14.3 (11.5 to 17.6)	299 (240 to 368)	1.11 (1.10 to 1.12)	<0.001
Australasia	15.8 (14.5 to 17.2)	708 (652 to 772)	51.6 (43.9 to 60.5)	1080 (919 to 1266)	1.47 (1.40 to 1.54)	<0.001
Caribbean	4.93 (4.05 to 5.93)	216 (178 to 260)	14.6 (11.7 to 17.9)	317 (256 to 389)	1.32 (1.31 to 1.34)	<0.001
Central Asia	12.5 (10.0 to 15.4)	359 (288 to 443)	25.8 (20.7 to 31.8)	514 (413 to 633)	1.25 (1.23 to 1.27)	<0.001
Central Europe	42.3 (35.0 to 50.6)	325 (268 to 389)	119 (95.6 to 146)	559 (450 to 686)	1.90 (1.83 to 1.96)	<0.001
Central Latin America	9.79 (7.87 to 12.0)	151 (121 to 184)	39.5 (31.7 to 48.4)	199 (159 to 244)	0.96 (0.95 to 0.98)	<0.001
Central Sub-Saharan Africa	4.40 (3.47 to 5.48)	269 (211 to 337)	12.5 (9.92 to 15.5)	357 (284 to 445)	0.98 (0.97 to 0.99)	<0.001
East Asia	68.5 (54.4 to 84.5)	98.8 (78.3 to 122)	340 (269 to 424)	184 (145 to 229)	2.17 (2.00 to 2.35)	<0.001
Eastern Europe	97.8 (78.7 to 121)	418 (335 to 516)	196 (157 to 241)	607 (486 to 749)	1.31 (1.27 to 1.35)	<0.001
Eastern Sub-Saharan Africa	15.4 (12.1 to 19.2)	274 (214 to 342)	38.9 (30.7 to 48.4)	336 (264 to 419)	0.70 (0.70 to 0.71)	<0.001
High-income Asia Pacific	69.2 (56.0 to 84.6)	397 (321 to 484)	213 (174 to 258)	477 (389 to 578)	0.64 (0.62 to 0.65)	<0.001
High-income North America	363 (298 to 435)	1051 (864 to 1261)	750 (602 to 930)	1248 (1003 to 1549)	0.60 (0.51 to 0.70)	<0.001
North Africa and Middle East	38.6 (31.1 to 47.1)	301 (242 to 368)	192 (154 to 234)	598 (480 to 731)	2.40 (2.36 to 2.43)	<0.001
Oceania	0.222 (0.177 to 0.274)	108 (85.8 to 134)	0.634 (0.503 to 0.785)	139 (110 to 172)	0.88 (0.87 to 0.88)	<0.001
South Asia	108 (84.8 to 133)	270 (212 to 336)	438 (347 to 544)	381 (300 to 474)	1.17 (1.09 to 1.25)	<0.001
South-East Asia Region	108 (85.0 to 133)	220 (173 to 273)	459 (364 to 569)	327 (259 to 406)	1.36 (1.29 to 1.43)	<0.001
Southern Latin America	14.6 (12.1 to 17.7)	355 (295 to 429)	38.4 (31.0 to 46.3)	498 (401 to 600)	1.19 (1.11 to 1.28)	<0.001
Southern Sub-Saharan Africa	7.92 (6.26 to 9.85)	361 (285 to 450)	19.2 (15.2 to 23.9)	437 (345 to 545)	0.66 (0.64 to 0.67)	<0.001
Tropical Latin America	26.7 (21.7 to 32.4)	373 (303 to 455)	124 (99.0 to 153)	598 (478 to 737)	1.64 (1.60 to 1.68)	<0.001
Western Europe	342 (293 to 397)	613 (525 to 712)	947 (764 to 1153)	1077 (868 to 1313)	1.95 (1.87 to 2.04)	<0.001

Western Africa	Sub-Saharan	19.9 (15.7 to 24.8)	293 (230 to 366)	47.4 (37.6 to 58.9)	361 (285 to 449)	0.71 (0.70 to 0.73)	<0.001

Note: Estimates are for individuals aged over 65 years. AAPCs=average annual percent changes. CI=confidence interval. P=P value for the significant test of AAPCs. SDI=Socio-demographic Index. Numbers in parentheses are 95% uncertainty intervals (Cases and age-standardized rate) and 95% confidence interval (AAPCs).

Supplementary Table 3. Age-standardized prevalence, mortality and DALYs of type 1 diabetes mellitus in elderly people in 2019 at regional levels by sex.

Regions	Age-standardized rate in 2019 (per 100,000)					
	Male			Female		
	Prevalence	Mortality	DALYs	Prevalence	Mortality	DALYs
Andean Latin America	275 (220 to 341)	2.73 (1.56 to 4.24)	80 (53 to 112)	321 (257 to 394)	3.68 (2.39 to 5.32)	97 (70 to 130)
Australasia	1126 (967 to 1317)	1.49 (0.882 to 3.13)	124 (85 to 174)	1038 (879 to 1224)	0.994 (0.565 to 2.09)	108 (73 to 153)
Caribbean	335 (269 to 414)	8.67 (5.81 to 13.2)	178 (129 to 255)	301 (242 to 368)	8.97 (5.89 to 13.3)	177 (126 to 244)
Central Asia	552 (443 to 682)	6.4 (4.02 to 9.97)	177 (125 to 248)	489 (393 to 601)	6.00 (3.78 to 9.58)	164 (116 to 232)
Central Europe	594 (478 to 729)	5.3 (3.82 to 9.17)	150 (115 to 217)	534 (429 to 655)	4.20 (2.99 to 6.62)	125 (96.0 to 164)
Central Latin America	198 (158 to 243)	3.88 (2.61 to 7.64)	90 (66 to 159)	199 (159 to 246)	3.67 (2.52 to 7.24)	84 (63 to 146)
Central Sub-Saharan Africa	360 (285 to 451)	7.88 (3.99 to 14)	171 (103 to 274)	355 (282 to 442)	3.85 (1.92 to 6.36)	100 (64 to 144)
East Asia	215 (169 to 270)	1.01 (0.757 to 1.44)	32 (25 to 42)	158 (125 to 196)	1.25 (0.952 to 1.69)	32 (26 to 41)
Eastern Europe	576 (460 to 712)	0.852 (0.541 to 2.3)	71 (48 to 105)	622 (498 to 766)	0.966 (0.551 to 3.35)	80 (53 to 128)
Eastern Sub-Saharan Africa	337 (264 to 421)	8.43 (4.41 to 13.7)	178 (110 to 265)	335 (263 to 416)	5.15 (3.03 to 7.13)	121 (84 to 157)
High-income Asia Pacific	501 (408 to 606)	0.679 (0.471 to 1.02)	54 (39 to 74)	456 (371 to 554)	0.41 (0.274 to 0.74)	48 (33 to 67)
High-income North America	1360 (1092 to 1688)	3.46 (1.76 to 5.09)	173 (125 to 229)	1156 (931 to 1434)	2.43 (1.37 to 3.90)	134 (98 to 180)
North Africa and Middle East	622 (499 to 762)	5.78 (3.54 to 7.78)	147 (107 to 189)	573 (460 to 701)	8.25 (5.92 to 11.2)	175 (136 to 223)
Oceania	145 (114 to 180)	13.9 (7.27 to 26.8)	245 (134 to 456)	133 (106 to 165)	5.77 (3.57 to 9.11)	109 (71 to 165)
South Asia	401 (316 to 501)	5.54 (3.46 to 9.89)	128 (91 to 201)	362 (286 to 449)	5.42 (1.51 to 8.2)	120 (53 to 167)
South-East Asia Region	348 (275 to 435)	6.42 (4.31 to 10.8)	138 (101 to 211)	308 (244 to 382)	6.98 (3.93 to 10.1)	139 (88 to 189)
Southern Latin America	519 (417 to 629)	4.62 (2.64 to 7.94)	129 (87 to 188)	482 (390 to 584)	3.25 (1.9 to 5.74)	98 (67 to 142)
Southern Sub-Saharan Africa	442 (348 to 551)	10.3 (7.59 to 13.5)	212 (164 to 266)	434 (342 to 540)	7.29 (5.54 to 9.7)	156 (125 to 199)
Tropical Latin America	449 (355 to 562)	5.42 (3.12 to 9.12)	134 (95 to 197)	711 (568 to 874)	5.32 (3.42 to 9.67)	152 (114 to 214)
Western Europe	1125 (905 to 1378)	2.16 (1.33 to 3.91)	119 (86 to 165)	1037 (836 to 1259)	1.54 (0.865 to 2.54)	101 (71 to 140)

Western Sub-Saharan Africa	360 (284 to 449)	8.3 (4.04 to 14.3)	177 (103 to 277)	361 (286 to 449)	5.76 (2.77 to 8.13)	135 (83 to 178)

Note: Estimates are for individuals aged over 65 years. AAPCs=average annual percent changes. CI=confidence interval. DALYs=disability-adjusted life years. P=P value for the significant test of AAPCs. Numbers in parentheses are 95% uncertainty intervals.

Supplementary Table 4. Age-standardized mortality and DALYs of type 1 diabetes mellitus in elderly people and their AAPCs from 1990 to 2019 at the regional levels.

Regions	Mortality						DALYs					
	Cases in 1990 (thousands)	Age-standardized rate in 1990 (per 100,000)	Cases in 2019 (thousands)	Age-standardized rate in 2019 (per 100,000)	AAPCs in rate, 1990-2019 (%/year)	P value	Cases in 1990 (thousands)	Age-standardized rate in 1990 (per 100,000)	Cases in 2019 (thousands)	Age-standardized rate in 2019 (per 100,000)	AAPCs in rate, 1990-2019 (%/year)	P value
Andean Latin America	0.0656 (0.0498 to 0.0853)	4.03 (3.06 to 5.26)	0.155 (0.106 to 0.212)	3.24 (2.21 to 4.42)	-0.77 (-1.07 to -0.47)	<0.001	1.58 (1.25 to 2.01)	96 (76 to 122)	4.27 (3.19 to 5.60)	89 (66 to 116)	-0.28 (-0.51 to -0.04)	0.02
Australasia	0.0561 (0.0375 to 0.0836)	2.52 (1.69 to 3.78)	0.0603 (0.0395 to 0.113)	1.23 (0.80 to 2.29)	-2.49 (-2.77 to -2.2)	<0.001	2.44 (1.84 to 3.15)	108 (82 to 140)	5.51 (3.81 to 7.63)	115 (80 to 160)	0.22 (0.09 to 0.35)	0.001
Caribbean	0.274 (0.190 to 0.353)	12.43 (8.64 to 16.16)	0.409 (0.297 to 0.559)	8.84 (6.43 to 12.08)	-1.13 (-1.32 to -0.94)	<0.001	5.26 (3.78 to 6.60)	229 (165 to 288)	8.16 (6.25 to 10.6)	177 (136 to 229)	-0.87 (-1.03 to -0.71)	<0.001
Central Asia	0.122 (0.0736 to 0.159)	3.43 (2.07 to 4.49)	0.330 (0.236 to 0.467)	6.16 (4.39 to 8.81)	2.13 (1.85 to 2.41)	<0.001	3.63 (2.55 to 4.60)	103 (72 to 130)	9.19 (6.99 to 12.1)	170 (129 to 225)	1.79 (1.58 to 2.00)	<0.001
Central Europe	1.06 (0.744 to 1.34)	8.02 (5.63 to 10.15)	0.989 (0.766 to 1.49)	4.68 (3.62 to 7.06)	-1.89 (-2.08 to -1.69)	<0.001	23.9 (17.8 to 29.1)	177 (133 to 215)	28.9 (23.0 to 37.3)	136 (108 to 176)	-0.92 (-1.07 to -0.77)	<0.001
Central Latin America	0.402 (0.245 to 0.529)	6.24 (3.79 to 8.19)	0.750 (0.566 to 1.31)	3.77 (2.84 to 6.61)	-1.76 (-1.98 to -1.53)	<0.001	8.36 (5.52 to 10.7)	126 (83 to 161)	17.3 (13.7 to 27.3)	87 (69 to 136)	-1.30 (-1.47 to -1.13)	<0.001
Central Sub-Saharan Africa	0.0957 (0.0479 to 0.160)	6.45 (3.18 to 10.87)	0.179 (0.104 to 0.272)	5.46 (3.16 to 8.35)	-0.57 (-0.61 to -0.53)	<0.001	2.31 (1.34 to 3.58)	139 (81 to 215)	4.60 (3.09 to 6.48)	130 (87 to 183)	-0.23 (-0.27 to -0.20)	<0.001
East Asia	1.22 (0.950 to	1.90 (1.47 to 2.35)	1.91 (1.56 to 2.42)	1.12 (0.91 to	-1.78 (-1.98 to -	<0.001	28.3 (22.7 to 34.2)	40 (32 to 48)	59.8 (48.3 to 74.6)	32 (26 to 40)	-0.74 (-0.91 to -	<0.001

	1.51)			1.45)	1.58)						0.57)	
Eastern Europe	0.300 (0.212 to 0.581)	1.24 (0.88 to 2.41)	0.298 (0.183 to 0.912)	0.93 (0.57 to 2.8)	-0.68 (-1.15 to -0.21)	0.005	15.4 (11.3 to 21.7)	65 (48 to 92)	24.9 (16.8 to 37.6)	77 (52 to 117)	0.66 (0.48 to 0.84)	<0.001
Eastern Sub-Saharan Africa	0.407 (0.201 to 0.656)	7.65 (3.76 to 12.37)	0.724 (0.433 to 0.990)	6.63 (3.93 to 9.09)	-0.50 (-0.58 to -0.41)	<0.001	9.37 (5.39 to 14.2)	162 (94 to 246)	17.3 (11.8 to 22.3)	147 (101 to 190)	-0.34 (-0.40 to -0.28)	<0.001
High-income Asia Pacific	0.312 (0.219 to 0.400)	1.77 (1.24 to 2.29)	0.247 (0.186 to 0.366)	0.53 (0.40 to 0.78)	-4.10 (-4.49 to -3.71)	<0.001	11.1 (8.57 to 14)	62 (48 to 79)	22.6 (16.1 to 31.0)	51 (36 to 70)	-0.69 (-0.78 to -0.61)	<0.001
High-income North America	1.30 (0.897 to 1.91)	3.74 (2.59 to 5.51)	1.76 (1.13 to 2.42)	2.90 (1.86 to 3.99)	-0.88 (-1.19 to -0.56)	<0.001	51.8 (40.1 to 66.7)	150 (116 to 193)	91.2 (67.9 to 119)	152 (113 to 199)	0.03 (-0.09 to 0.15)	0.59
North Africa and Middle East	1.15 (0.833 to 1.49)	10.15 (7.16 to 13.31)	2.06 (1.52 to 2.57)	7.03 (5.10 to 8.81)	-1.26 (-1.36 to -1.17)	<0.001	23.5 (18 to 29.4)	186 (140 to 234)	51.2 (40.6 to 62.9)	161 (127 to 199)	-0.48 (-0.57 to -0.39)	<0.001
Oceania	0.0197 (0.0122 to 0.0342)	10.52 (6.48 to 18.10)	0.0417 (0.0260 to 0.0715)	9.74 (6.10 to 16.58)	-0.27 (-0.34 to -0.20)	<0.001	0.398 (0.256 to 0.679)	187 (120 to 317)	0.837 (0.538 to 1.40)	178 (115 to 295)	-0.18 (-0.24 to -0.12)	<0.001
South Asia	1.91 (0.638 to 3.06)	5.36 (1.78 to 8.67)	5.85 (3.08 to 8.83)	5.48 (2.85 to 8.28)	0.03 (-0.52 to 0.59)	0.91	45.5 (21.2 to 66.0)	114 (53 to 166)	143 (91.9 to 199)	124 (79 to 172)	0.28 (-0.16 to 0.72)	0.21
South-East Asia Region	3.13 (1.51 to 4.77)	7.38 (3.57 to 11.24)	8.74 (5.97 to 12.6)	6.74 (4.61 to 9.68)	-0.33 (-0.61 to -0.04)	0.03	68.2 (37.8 to 98.8)	141 (78 to 204)	195 (146 to 267)	139 (104 to 189)	-0.07 (-0.34 to 0.20)	0.63
Southern Latin America	0.300 (0.171 to 0.400)	7.19 (4.12 to 9.65)	0.299 (0.199 to 0.473)	3.86 (2.58 to 6.1)	-2.10 (-2.46 to -1.75)	<0.001	6.74 (4.29 to 8.61)	159 (102 to 204)	8.60 (6.34 to 11.7)	112 (82 to 152)	-1.20 (-1.44 to -0.96)	<0.001
Southern Sub-	0.154 (0.121 to	7.60 (5.95 to 9.32)	0.341 (0.278 to	8.44 (6.83 to	0.39 (-0.09 to	0.11	3.47 (2.84 to 4.15)	158 (130 to 190)	7.83 (6.53 to 9.44)	178 (149 to 215)	0.44 (0.01 to	0.04

Saharan Africa	0.188)		0.418)	10.38)	0.88)						0.86)	
Tropical Latin America	0.582 (0.360 to 0.801)	9.01 (5.54 to 12.47)	1.10 (0.801 to 1.74)	5.41 (3.94 to 8.57)	-1.70 (-1.82 to -1.58)	<0.001	12.7 (8.91 to 16.5)	179 (125 to 233)	29.9 (23.4 to 40.3)	144 (113 to 195)	-0.71 (-0.83 to -0.59)	<0.001
Western Europe	2.23 (1.33 to 3.08)	4.03 (2.39 to 5.56)	1.77 (1.22 to 2.69)	1.83 (1.28 to 2.82)	-2.71 (-2.90 to -2.51)	<0.001	60.1 (44.4 to 76.5)	107 (79 to 137)	96.7 (69.4 to 131)	109 (78 to 149)	0.05 (-0.07 to 0.18)	0.4
Western Sub-Saharan Africa	0.613 (0.302 to 0.989)	9.43 (4.61 to 15.17)	0.882 (0.473 to 1.31)	6.96 (3.70 to 10.39)	-1.04 (-1.12 to -0.96)	<0.001	13.4 (7.53 to 20.5)	192 (108 to 294)	20.8 (13.1 to 28.6)	155 (98 to 214)	-0.73 (-0.79 to -0.68)	<0.001

Note: Estimates are for individuals aged over 65 years. AAPCs=average annual percent changes. CI=confidence interval. DALYs=disability-adjusted life years. P=P value for the significant test of AAPCs. Numbers in parentheses are 95% uncertainty intervals (Cases and age-standardized rate) and 95% confidence interval (AAPCs).

Supplementary Table 5. Age-standardized prevalence, mortality, and DALYs of type 1 diabetes mellitus in elderly people in 2019 and their AAPCs between 1990-2019 in 204 countries and territories.

	Age-standardized rate in 2019 (per 100,000)			AAPCs 1990-2019 (%/year)		
	**Prevalence**	**Mortality**	**DALYs**	**Prevalence**	**Mortality**	**DALYs**
Afghanistan	397 (319 to 487)	11.1 (5.22 to 20.3)	216 (119 to 360)	1.69 (1.62 to 1.76)	-0.57 (-0.69 to -0.46)	-0.38 (-0.49 to -0.27)
Albania	477 (383 to 593)	3.71 (2.16 to 6.25)	112 (78 to 161)	1.31 (1.30 to 1.33)	-1.53 (-1.89 to -1.18)	-0.64 (-0.91 to -0.38)
Algeria	560 (448 to 688)	6.25 (3.53 to 10.2)	137 (93 to 195)	2.73 (2.67 to 2.80)	-0.91 (-1.06 to -0.76)	-0.12 (-0.20 to -0.03)
American Samoa	172 (137 to 212)	6.03 (3.91 to 8.84)	115 (79 to 160)	0.95 (0.93 to 0.97)	0.59 (0.18 to 0.99)	0.63 (0.26 to 1.00)
Andorra	1241 (1012 to 1519)	3.10 (1.78 to 4.95)	143 (100 to 201)	1.99 (1.96 to 2.02)	-0.87 (-1.00 to -0.74)	0.74 (0.67 to 0.82)
Angola	338 (269 to 418)	6.07 (3.21 to 9.98)	138 (87 to 205)	0.98 (0.96 to 1.00)	-0.37 (-0.44 to -0.3)	-0.20 (-0.26 to -0.14)
Antigua and Barbuda	322 (258 to 397)	16.9 (10.9 to 27.7)	297 (204 to 461)	1.38 (1.37 to 1.39)	-0.67 (-1.57 to 0.23)	-0.65 (-1.56 to 0.26)
Argentina	471 (378 to 571)	4.64 (2.90 to 7.31)	125 (88 to 173)	1.44 (1.35 to 1.53)	-2.09 (-2.53 to -1.64)	-1.28 (-1.70 to -0.87)
Armenia	522 (421 to 645)	5.75 (3.56 to 9.96)	157 (110 to 235)	1.24 (1.22 to 1.27)	0.07 (-1.09 to 1.24)	0.31 (-0.52 to 1.15)
Australia	1073 (922 to 1252)	1.29 (0.824 to 2.39)	116 (79 to 163)	1.75 (1.70 to 1.81)	-2.06 (-2.45 to -1.66)	0.51 (0.39 to 0.62)
Austria	996 (807 to 1216)	1.88 (1.22 to 3.12)	117 (81 to 164)	2.83 (2.75 to 2.90)	-2.12 (-2.58 to -1.66)	0.74 (0.60 to 0.88)
Azerbaijan	477 (383 to 590)	6.90 (4.21 to 11.1)	177 (122 to 259)	1.17 (1.16 to 1.18)	2.11 (1.89 to 2.34)	1.75 (1.54 to 1.97)
Bahamas	359 (288 to 441)	9.81 (6.12 to 15.9)	198 (136 to 295)	1.57 (1.55 to 1.58)	-1.30 (-1.59 to -1.02)	-0.96 (-1.33 to -0.59)
Bahrain	667 (535 to 813)	7.46 (4.45 to 10.9)	163 (114 to 219)	2.59 (2.53 to 2.66)	-1.34 (-2.00 to -0.68)	-0.58 (-1.22 to 0.07)
Bangladesh	356 (285 to 437)	6.64 (3.08 to 11.4)	128 (76 to 197)	1.10 (1.08 to 1.11)	-0.11 (-0.77 to 0.54)	-0.05 (-0.53 to 0.43)
Barbados	233 (188 to 288)	14.7 (9.06 to 24.0)	255 (166 to 391)	1.48 (1.45 to 1.50)	-0.98 (-1.48 to -0.48)	-0.91 (-1.38 to -0.43)
Belarus	512 (413 to 632)	0.54 (0.282 to 0.834)	60 (40 to 87)	1.74 (1.72 to 1.77)	-2.54 (-3.31 to -1.76)	0.45 (0.22 to 0.68)
Belgium	917 (744 to 1115)	1.75 (1.11 to 2.75)	110 (76 to 154)	2.13 (2.02 to 2.24)	-2.83 (-3.17 to -2.48)	-0.0009 (-0.13 to 0.13)
Belize	279 (224 to 346)	9.43 (6.06 to 16.1)	185 (129 to 297)	1.21 (1.20 to 1.22)	-0.82 (-1.46 to -0.17)	-0.52 (-1.09 to 0.06)
Benin	297 (235 to 371)	6.84 (3.13 to 12.1)	148 (83 to 240)	0.50 (0.49 to 0.51)	-1.01 (-1.12 to -0.91)	-0.77 (-0.88 to -0.66)
Bermuda	433 (348 to 534)	5.46 (3.29 to 9.08)	129 (90 to 187)	2.11 (2.10 to 2.12)	-2.9 (-3.13 to -2.67)	-1.96 (-2.11 to -1.82)
Bhutan	337 (269 to 417)	6.50 (3.67 to 11.4)	137 (89 to 216)	1.06 (1.05 to 1.07)	0.60 (0.53 to 0.67)	0.56 (0.50 to 0.63)
Bolivia (Plurinational State of)	287 (229 to 354)	6.84 (3.81 to 11.0)	153 (97 to 228)	1.18 (1.13 to 1.23)	-0.61 (-0.70 to -0.53)	-0.41 (-0.49 to -0.33)
Bosnia and Herzegovina	499 (400 to 616)	9.02 (5.45 to 13.4)	203 (140 to 281)	2.14 (2.09 to 2.19)	-1.73 (-2.27 to -1.18)	-1.28 (-1.71 to -0.86)
Botswana	399 (319 to 494)	10.3 (5.67 to 16.9)	208 (131 to 316)	0.84 (0.81 to 0.87)	-0.56 (-0.63 to -0.48)	-0.37 (-0.44 to -0.31)

Brazil	601 (480 to 742)	5.41 (3.93 to 8.66)	145 (113 to 196)	1.64 (1.60 to 1.68)	-1.74 (-1.86 to -1.61)	-0.73 (-0.85 to -0.61)
Brunei Darussalam	442 (355 to 536)	11.9 (7.95 to 17.2)	234 (168 to 324)	0.28 (0.27 to 0.29)	-2.44 (-2.96 to -1.92)	-2.27 (-2.75 to -1.79)
Bulgaria	546 (438 to 673)	11.4 (5.66 to 17.5)	248 (150 to 352)	1.80 (1.78 to 1.83)	0.85 (0.56 to 1.14)	0.89 (0.68 to 1.09)
Burkina Faso	283 (224 to 352)	7.02 (3.20 to 12.4)	147 (82 to 237)	0.37 (0.36 to 0.38)	-1.42 (-1.66 to -1.19)	-1.14 (-1.31 to -0.98)
Burundi	315 (249 to 393)	6.64 (3.02 to 11.8)	146 (84 to 240)	0.69 (0.67 to 0.70)	-0.82 (-0.88 to -0.76)	-0.62 (-0.68 to -0.56)
Cabo Verde	361 (289 to 448)	6.19 (3.08 to 11.4)	142 (86 to 232)	0.94 (0.93 to 0.95)	0.70 (-0.01 to 1.40)	0.64 (0.10 to 1.19)
Cambodia	112 (89.4 to 138)	11.2 (5.98 to 21.6)	187 (106 to 352)	0.85 (0.83 to 0.86)	-0.31 (-0.41 to -0.21)	-0.37 (-0.48 to -0.26)
Cameroon	348 (276 to 434)	7.66 (3.94 to 13.1)	168 (101 to 264)	0.76 (0.74 to 0.79)	-0.88 (-1.00 to -0.76)	-0.61 (-0.74 to -0.48)
Canada	1680 (1367 to 2062)	4.42 (2.59 to 6.54)	211 (150 to 288)	1.08 (0.80 to 1.37)	-2.00 (-2.33 to -1.66)	-0.33 (-0.52 to -0.13)
Central African Republic	278 (220 to 348)	8.38 (4.01 to 14.7)	177 (99 to 289)	0.53 (0.51 to 0.55)	0.01 (-0.07 to 0.08)	0.04 (-0.03 to 0.10)
Chad	270 (213 to 339)	6.65 (2.63 to 12.7)	144 (73 to 250)	0.36 (0.35 to 0.38)	-0.59 (-0.73 to -0.45)	-0.41 (-0.52 to -0.29)
Chile	459 (369 to 556)	2.28 (1.45 to 4.11)	77 (55 to 111)	1.48 (1.43 to 1.52)	-1.98 (-2.52 to -1.44)	-0.79 (-1.25 to -0.33)
China	181 (143 to 226)	0.881 (0.707 to 1.11)	28 (23 to 36)	2.16 (1.97 to 2.34)	-1.96 (-2.21 to -1.71)	-0.67 (-0.93 to -0.41)
Colombia	242 (194 to 296)	1.00 (0.574 to 1.90)	43 (29 to 61)	1.18 (1.17 to 1.19)	-3.23 (-3.64 to -2.81)	-1.51 (-1.66 to -1.35)
Comoros	362 (287 to 450)	7.17 (3.53 to 12.1)	158 (95 to 242)	1.03 (1.00 to 1.05)	-0.16 (-0.26 to -0.05)	0.02 (-0.09 to 0.14)
Congo	404 (320 to 502)	7.58 (3.91 to 13.1)	169 (103 to 263)	1.25 (1.24 to 1.26)	-0.30 (-0.54 to -0.06)	-0.09 (-0.33 to 0.15)
Cook Islands	192 (153 to 237)	6.19 (4.14 to 9.01)	115 (83 to 160)	0.93 (0.91 to 0.95)	-1.70 (-1.88 to -1.52)	-1.52 (-1.67 to -1.36)
Costa Rica	209 (168 to 258)	0.855 (0.49 to 1.76)	36 (25 to 53)	0.74 (0.72 to 0.75)	-2.40 (-4.06 to -0.71)	-1.03 (-1.72 to -0.34)
Côte d'Ivoire	306 (243 to 379)	7.42 (3.72 to 12.9)	159 (93 to 253)	0.56 (0.55 to 0.57)	-1.06 (-1.19 to -0.93)	-0.81 (-0.91 to -0.70)
Croatia	690 (554 to 848)	3.75 (2.26 to 6.34)	130 (93 to 178)	2.49 (2.39 to 2.59)	-1.94 (-2.39 to -1.48)	-0.48 (-0.97 to 0.001)
Cuba	255 (204 to 317)	1.3 (0.764 to 2.73)	49 (34 to 73)	1.64 (1.56 to 1.71)	-4.25 (-4.99 to -3.51)	-2.40 (-2.69 to -2.11)
Cyprus	1085 (873 to 1326)	4.85 (1.75 to 7.77)	155 (96 to 218)	3.04 (2.97 to 3.11)	-3.34 (-3.62 to -3.06)	-1.34 (-1.60 to -1.07)
Czechia	710 (571 to 870)	6.93 (4.05 to 14.4)	177 (123 to 294)	1.83 (1.79 to 1.87)	-1.73 (-2.32 to -1.13)	-0.98 (-1.20 to -0.75)
Democratic People's Republic of Korea	178 (143 to 219)	1.96 (0.978 to 4.02)	51 (32 to 86)	1.60 (1.56 to 1.63)	-0.94 (-1.01 to -0.86)	-0.32 (-0.38 to -0.26)
Democratic Republic of the Congo	362 (286 to 452)	4.91 (2.5 to 8.49)	120 (76 to 184)	0.97 (0.95 to 0.99)	-0.74 (-0.83 to -0.64)	-0.3 (-0.38 to -0.23)
Denmark	1043 (844 to 1271)	3.61 (2.33 to 6.01)	128 (93 to 176)	1.84 (1.81 to 1.88)	-1.77 (-2.20 to -1.33)	-0.34 (-0.59 to -0.09)
Djibouti	346 (273 to 431)	6.80 (3.25 to 11.8)	151 (90 to 236)	0.92 (0.91 to 0.93)	0.06 (-0.04 to 0.15)	0.18 (0.09 to 0.27)
Dominica	355 (285 to 438)	12.7 (7.91 to 19.5)	241 (163 to 347)	1.36 (1.33 to 1.39)	-0.81 (-0.98 to -0.64)	-0.60 (-0.90 to -0.31)
Dominican	272 (219 to 334)	9.64 (5.57 to 15.5)	183 (118 to 271)	1.22 (1.22 to 1.23)	0.45 (-0.04 to 0.95)	0.59 (0.03 to 1.15)

Republic						
Ecuador	296 (238 to 365)	2.97 (1.91 to 4.52)	82 (59 to 112)	1.12 (1.1 to 1.13)	-1.23 (-1.92 to -0.54)	-0.53 (-0.99 to -0.07)
Egypt	528 (425 to 651)	8.46 (4.74 to 14.6)	186 (123 to 283)	1.67 (1.64 to 1.70)	-1.39 (-1.78 to -1)	-0.8 (-1.00 to -0.60)
El Salvador	188 (151 to 232)	4.72 (2.60 to 7.32)	105 (65 to 154)	0.95 (0.95 to 0.96)	0.06 (-0.70 to 0.82)	0.08 (-0.67 to 0.83)
Equatorial Guinea	407 (324 to 503)	6.23 (3.39 to 10.4)	144 (93 to 217)	1.42 (1.40 to 1.43)	0.02 (-0.16 to 0.20)	0.14 (-0.0034 to 0.29)
Eritrea	325 (255 to 405)	8.00 (3.58 to 13.6)	171 (96 to 267)	0.90 (0.89 to 0.91)	0.42 (0.30 to 0.54)	0.36 (0.24 to 0.48)
Estonia	681 (546 to 838)	2.13 (1.17 to 3.41)	106 (72 to 147)	1.73 (1.68 to 1.78)	-1.98 (-3.62 to -0.31)	-0.01 (-0.89 to 0.88)
Eswatini	366 (292 to 452)	11.7 (6.16 to 19.7)	228 (137 to 361)	0.66 (0.65 to 0.66)	-0.30 (-0.48 to -0.13)	-0.22 (-0.37 to -0.06)
Ethiopia	324 (252 to 407)	6.63 (3.43 to 10.2)	145 (91 to 203)	0.72 (0.71 to 0.74)	-1.02 (-1.12 to -0.92)	-0.91 (-1.06 to -0.76)
Fiji	168 (134 to 209)	14.8 (9.94 to 21.5)	258 (180 to 364)	1.15 (1.14 to 1.16)	-0.95 (-1.27 to -0.62)	-0.91 (-1.21 to -0.61)
Finland	1693 (1382 to 2072)	1.56 (0.926 to 3.26)	156 (103 to 222)	0.44 (0.21 to 0.67)	-5.20 (-5.57 to -4.83)	-1.32 (-1.69 to -0.95)
France	884 (714 to 1080)	2.67 (1.37 to 4.15)	98 (69 to 134)	3.16 (3.1 to 3.22)	-2.44 (-2.77 to -2.12)	0.10 (-0.07 to 0.26)
Gabon	403 (322 to 496)	7.27 (3.98 to 11.9)	164 (104 to 244)	1.19 (1.18 to 1.20)	-0.09 (-0.26 to 0.08)	0.12 (0.0026 to 0.23)
Gambia	327 (258 to 408)	8.30 (3.80 to 15.5)	176 (97 to 300)	0.79 (0.78 to 0.80)	0.14 (-0.30 to 0.58)	0.27 (-0.15 to 0.70)
Georgia	561 (449 to 692)	10.2 (4.23 to 15.6)	248 (132 to 352)	1.30 (1.26 to 1.33)	1.42 (0.80 to 2.04)	1.31 (0.86 to 1.77)
Germany	912 (735 to 1105)	1.84 (1.19 to 3.44)	94 (66 to 131)	2.67 (2.61 to 2.74)	-3.68 (-4.02 to -3.34)	-0.83 (-0.97 to -0.69)
Ghana	387 (307 to 480)	6.04 (3.37 to 9.97)	143 (94 to 213)	0.80 (0.78 to 0.82)	0.21 (0.08 to 0.35)	0.36 (0.24 to 0.49)
Greece	958 (774 to 1166)	0.356 (0.214 to 0.747)	91 (58 to 136)	3.16 (2.94 to 3.38)	-1.75 (-2.3 to -1.2)	2.49 (2.23 to 2.75)
Greenland	1034 (833 to 1267)	0.774 (0.454 to 1.2)	99 (66 to 143)	0.59 (0.57 to 0.62)	-1.89 (-2.65 to -1.13)	0.05 (-0.04 to 0.15)
Grenada	310 (249 to 380)	11.9 (7.74 to 19.8)	231 (161 to 361)	1.43 (1.42 to 1.45)	-0.97 (-1.71 to -0.22)	-0.75 (-1.48 to -0.01)
Guam	189 (150 to 232)	2.24 (1.37 to 3.48)	55 (38 to 77)	0.77 (0.75 to 0.78)	-0.72 (-1.46 to 0.03)	-0.09 (-0.57 to 0.38)
Guatemala	179 (143 to 221)	7.58 (3.46 to 11.9)	153 (77 to 228)	0.90 (0.88 to 0.91)	2.65 (1.87 to 3.44)	2.26 (1.38 to 3.14)
Guinea	285 (225 to 355)	8.37 (4.05 to 14.5)	175 (98 to 282)	0.41 (0.40 to 0.42)	-0.66 (-0.73 to -0.60)	-0.51 (-0.56 to -0.46)
Guinea-Bissau	298 (235 to 374)	9.55 (4.75 to 16.3)	199 (114 to 321)	0.60 (0.59 to 0.61)	-0.82 (-0.90 to -0.74)	-0.69 (-0.77 to -0.61)
Guyana	283 (226 to 350)	16.2 (10.3 – 27.0)	299 (203 to 477)	1.22 (1.21 to 1.23)	-1.12 (-1.63 to -0.61)	-1.00 (-1.49 to -0.50)
Haiti	264 (211 to 328)	20.3 (10.5 – 33.0)	369 (203 to 582)	1.42 (1.42 to 1.43)	-0.73 (-0.79 to -0.67)	-0.69 (-0.76 to -0.62)
Honduras	178 (142 to 219)	3.98 (1.27 to 7.42)	92 (39 to 155)	0.75 (0.74 to 0.76)	1.29 (0.74 to 1.85)	1.12 (0.69 to 1.55)
Hungary	640 (514 to 787)	2.91 (1.66 to 6.44)	113 (78 to 176)	2.09 (2.02 to 2.15)	-2.04 (-2.61 to -1.46)	-0.43 (-0.81 to -0.05)
Iceland	759 (622 to 919)	1.65 (1.01 to 2.77)	84 (59 to 118)	1.41 (1.26 to 1.55)	-2.18 (-2.47 to -1.90)	-0.21 (-0.36 to -0.05)
India	386 (304 to 482)	5.3 (2.7 to 8.15)	123 (78 to 172)	1.25 (1.16 to 1.34)	0.05 (-0.66 to 0.78)	0.25 (-0.02 to 0.53)
Indonesia	151 (120 to 189)	13.1 (8.6 to 23.8)	228 (155 to 406)	1.28 (1.28 to 1.29)	0.49 (0.40 to 0.58)	0.41 (0.33 to 0.49)
Iran (Islamic Republic of)	629 (503 to 773)	4.27 (3.28 to 5.12)	124 (99 to 156)	2.29 (2.25 to 2.34)	-1.68 (-1.85 to -1.50)	-0.40 (-0.55 to -0.26)
Iraq	561 (450 to 693)	3.59 (2.11 to 6.02)	105 (74 to 148)	2.23 (2.19 to 2.27)	-0.32 (-0.62 to -0.01)	0.50 (0.27 to 0.72)

Ireland	1265 (1025 to 1549)	1.94 (1.18 to 3.07)	128 (88 to 181)	2.37 (2.27 to 2.46)	-2.81 (-3.19 to -2.43)	0.08 (-0.38 to 0.54)
Israel	666 (590 to 759)	2.78 (1.5 to 4.11)	97 (68 to 129)	1.02 (0.94 to 1.09)	-0.39 (-1.01 to 0.24)	0.23 (0.05 to 0.41)
Italy	1276 (1025 to 1571)	2.21 (1.36 to 3.36)	131 (93 to 177)	1.15 (0.92 to 1.37)	-0.59 (-0.82 to -0.36)	0.76 (0.55 to 0.97)
Jamaica	340 (273 to 416)	9.97 (5.71 to 21.8)	195 (126 to 380)	1.52 (1.50 to 1.53)	-2.00 (-3.87 to -0.09)	-1.66 (-3.24 to -0.05)
Japan	469 (381 to 570)	0.312 (0.229 to 0.575)	48 (33 to 66)	0.59 (0.55 to 0.63)	-4.6 (-4.84 to -4.36)	-0.31 (-0.35 to -0.27)
Jordan	439 (351 to 538)	6.04 (3.35 to 9.23)	125 (84 to 174)	2.73 (2.67 to 2.79)	-1.89 (-2.29 to -1.49)	-1.06 (-1.42 to -0.70)
Kazakhstan	536 (430 to 659)	2.33 (1.17 to 6.76)	96 (61 to 178)	1.34 (1.31 to 1.36)	-0.52 (-1.72 to 0.69)	0.3 (-0.33 to 0.94)
Kenya	381 (298 to 477)	5.9 (3.69 to 8.18)	140 (99 to 182)	0.59 (0.58 to 0.60)	0.25 (0.12 to 0.37)	0.34 (0.26 to 0.42)
Kiribati	152 (120 to 189)	9.38 (5.16 to 16.1)	170 (101 to 280)	1.04 (1.03 to 1.06)	-0.36 (-0.43 to -0.29)	-0.41 (-0.47 to -0.35)
Kuwait	1060 (856 to 1295)	0.342 (0.193 to 0.53)	101 (64 to 149)	1.92 (1.81 to 2.03)	-2.41 (-4.90 to 0.15)	1.45 (0.97 to 1.94)
Kyrgyzstan	517 (414 to 638)	0.774 (0.486 to 1.47)	67 (45 to 95)	1.24 (1.23 to 1.25)	-0.31 (-1.03 to 0.41)	0.78 (0.63 to 0.93)
Lao People's Democratic Republic	109 (86.4 to 135)	10.2 (5.04 to 21.2)	172 (90 to 351)	0.93 (0.91 to 0.94)	-0.89 (-0.95 to -0.83)	-0.95 (-1.02 to -0.88)
Latvia	494 (398 to 608)	0.537 (0.321 to 1.09)	58 (38 to 84)	2.24 (2.23 to 2.25)	-1.93 (-2.53 to -1.32)	1.03 (0.79 to 1.27)
Lebanon	616 (494 to 753)	4.75 (2.5 to 8.25)	132 (89 to 192)	2.2 (2.17 to 2.23)	-2.45 (-2.52 to -2.38)	-1.18 (-1.28 to -1.08)
Lesotho	363 (287 to 452)	14.0 (7.42 to 23.4)	272 (160 to 432)	0.56 (0.55 to 0.58)	0.73 (0.55 to 0.9)	0.67 (0.58 to 0.76)
Liberia	337 (266 to 422)	7.12 (3.14 to 13.2)	156 (86 to 261)	0.77 (0.75 to 0.8)	-1.31 (-1.53 to -1.08)	-1.01 (-1.19 to -0.83)
Libya	832 (667 to 1018)	6.65 (3.67 to 11)	180 (124 to 254)	3.28 (3.26 to 3.3)	-0.47 (-0.94 to 0.01)	0.55 (0.24 to 0.86)
Lithuania	488 (394 to 603)	0.894 (0.528 to 1.82)	65 (43 to 93)	2.07 (2.03 to 2.11)	-3.00 (-3.53 to -2.46)	-0.17 (-0.45 to 0.12)
Luxembourg	1006 (813 to 1223)	2.01 (1.23 to 3.26)	108 (75 to 150)	2.33 (2.19 to 2.46)	-3.27 (-3.44 to -3.09)	-0.40 (-0.51 to -0.28)
Madagascar	336 (266 to 418)	5.25 (2.16 to 9.43)	125 (70 to 198)	0.67 (0.66 to 0.68)	-0.42 (-0.58 to -0.25)	-0.16 (-0.30 to -0.01)
Malawi	332 (261 to 414)	6.98 (3.73 to 11.5)	153 (96 to 231)	0.70 (0.69 to 0.70)	-0.36 (-0.49 to -0.24)	-0.18 (-0.27 to -0.08)
Malaysia	154 (123 to 190)	13.3 (8.22 to 21)	231 (150 to 354)	1.18 (1.08 to 1.28)	-2.15 (-2.86 to -1.42)	-2.21 (-2.73 to -1.68)
Maldives	129 (103 to 159)	7.3 (4.46 to 11.2)	125 (82 to 185)	1.22 (1.21 to 1.24)	-1.57 (-1.89 to -1.25)	-1.70 (-2.03 to -1.37)
Mali	283 (224 to 353)	7.15 (3.1 to 12.6)	153 (82 to 247)	0.39 (0.37 to 0.41)	-1.10 (-1.29 to -0.92)	-0.89 (-1.02 to -0.76)
Malta	1279 (1032 to 1566)	2.69 (1.63 to 4.14)	141 (98 to 194)	1.74 (1.69 to 1.80)	-3.11 (-3.36 to -2.85)	-0.58 (-0.79 to -0.37)
Marshall Islands	149 (118 to 185)	9.39 (5.32 to 15.3)	168 (101 to 265)	1.04 (1.02 to 1.06)	-0.42 (-0.56 to -0.27)	-0.42 (-0.55 to -0.28)
Mauritania	365 (289 to 454)	5.82 (1.98 to 11.1)	136 (68 to 224)	0.86 (0.86 to 0.87)	-1.68 (-1.90 to -1.45)	-1.26 (-1.49 to -1.03)
Mauritius	145 (116 to 178)	18.7 (11.5 to 35.1)	332 (211 to 592)	1.42 (1.37 to 1.48)	0.17 (-0.42 to 0.77)	0.11 (-0.81 to 1.03)
Mexico	180 (144 to 223)	5.18 (3.73 to 9.71)	108 (83 to 188)	0.86 (0.82 to 0.90)	-1.93 (-2.27 to -1.59)	-1.64 (-1.91 to -1.37)
Micronesia (Federated States of)	159 (126 to 197)	9.72 (5.55 to 16.1)	175 (105 to 280)	0.88 (0.86 to 0.91)	-0.36 (-0.4 to -0.31)	-0.34 (-0.38 to -0.30)

Monaco	1151 (927 to 1404)	1.87 (1.11 to 2.95)	118 (81 to 166)	1.70 (1.62 to 1.78)	-2.00 (-2.07 to -1.94)	0.35 (0.29 to 0.42)
Mongolia	492 (393 to 605)	1.45 (0.782 to 2.34)	76 (52 to 108)	1.50 (1.48 to 1.51)	-1.79 (-2.46 to -1.11)	-0.20 (-0.59 to 0.19)
Montenegro	661 (532 to 813)	11.4 (7.10 – 18.0)	260 (181 to 373)	1.65 (1.61 to 1.69)	0.12 (-0.27 to 0.50)	0.43 (0.13 to 0.72)
Morocco	540 (435 to 659)	8.57 (4.84 to 13.9)	181 (120 to 264)	2.18 (2.14 to 2.22)	-0.01 (-0.35 to 0.32)	0.39 (0.25 to 0.52)
Mozambique	298 (237 to 370)	7.75 (4.38 to 12.4)	163 (104 to 242)	0.59 (0.58 to 0.60)	0.04 (-0.08 to 0.16)	0.12 (0.03 to 0.21)
Myanmar	124 (98.8 to 153)	17.1 (9.11 to 34.2)	274 (151 to 546)	1.07 (1.06 to 1.08)	-0.87 (-0.93 to -0.81)	-1.06 (-1.13 to -1.00)
Namibia	395 (314 to 490)	9.96 (5.36 to 15.9)	201 (125 to 298)	0.69 (0.68 to 0.70)	-0.76 (-0.87 to -0.64)	-0.60 (-0.71 to -0.50)
Nauru	161 (128 to 198)	8.42 (5.04 to 13.6)	153 (98 to 238)	0.74 (0.73 to 0.75)	-0.40 (-0.48 to -0.33)	-0.33 (-0.40 to -0.26)
Nepal	320 (256 to 395)	3.95 (1.90 to 6.95)	89 (56 to 135)	0.87 (0.85 to 0.88)	0.76 (0.69 to 0.83)	0.68 (0.62 to 0.73)
Netherlands	1030 (832 to 1258)	3.04 (1.93 to 4.67)	134 (95 to 184)	2.11 (2.02 to 2.20)	-3.78 (-4.07 to -3.49)	-1.29 (-1.45 to -1.14)
New Zealand	1113 (895 to 1357)	0.867 (0.552 to 1.77)	114 (77 to 158)	0.38 (0.29 to 0.47)	-4.68 (-5.05 to -4.30)	-0.91 (-1.06 to -0.77)
Nicaragua	184 (148 to 227)	3.44 (2.32 to 5.01)	79 (58 to 107)	0.88 (0.87 to 0.88)	0.27 (-0.19 to 0.73)	0.33 (-0.33 to 0.99)
Niger	266 (210 to 332)	6.32 (2.13 to 11.5)	137 (64 to 229)	0.36 (0.35 to 0.37)	-0.83 (-0.93 to -0.73)	-0.66 (-0.75 to -0.57)
Nigeria	413 (325 to 515)	6.80 (3.15 to 11.8)	156 (93 to 239)	0.90 (0.87 to 0.92)	-1.26 (-1.35 to -1.16)	-0.86 (-0.92 to -0.80)
Niue	205 (163 to 252)	5.87 (3.47 to 9.6)	115 (75 to 177)	1.14 (1.12 to 1.16)	-0.76 (-0.86 to -0.65)	-0.53 (-0.58 to -0.48)
North Macedonia	540 (433 to 664)	13.2 (8.15 to 20.3)	277 (188 to 401)	1.66 (1.64 to 1.67)	-0.45 (-0.62 to -0.28)	-0.27 (-0.49 to -0.05)
Northern Mariana Islands	171 (137 to 211)	5.02 (3.13 to 7.56)	99 (67 to 142)	0.71 (0.59 to 0.83)	0.50 (0.26 to 0.75)	0.56 (0.21 to 0.92)
Norway	1414 (1134 to 1754)	2.14 (1.57 to 4.28)	142 (101 to 196)	0.76 (0.71 to 0.82)	-1.60 (-1.94 to -1.26)	-0.01 (-0.12 to 0.10)
Oman	659 (527 to 806)	24.7 (14.7 to 38.3)	418 (275 to 613)	2.61 (2.55 to 2.67)	-0.55 (-1.04 to -0.06)	-0.52 (-0.91 to -0.12)
Pakistan	360 (282 to 449)	6.12 (2.66 to 10.3)	139 (79 to 211)	0.71 (0.69 to 0.74)	0.72 (0.65 to 0.79)	0.70 (0.64 to 0.77)
Palau	197 (157 to 244)	5.28 (2.93 to 8.97)	104 (65 to 165)	1.09 (1.06 to 1.11)	-0.53 (-0.59 to -0.47)	-0.35 (-0.44 to -0.27)
Palestine	581 (466 to 715)	11.0 (6.99 – 16.0)	220 (156 to 296)	2.05 (2.01 to 2.10)	-1.03 (-1.33 to -0.73)	-0.71 (-1.04 to -0.37)
Panama	216 (173 to 265)	4.89 (2.35 to 7.60)	99 (61 to 142)	0.90 (0.89 to 0.91)	0.31 (-0.31 to 0.94)	0.30 (-0.16 to 0.77)
Papua New Guinea	126 (99.7 to 157)	9.95 (4.99 to 19.3)	180 (96 to 339)	0.81 (0.80 to 0.81)	0.14 (0.06 to 0.22)	0.15 (0.06 to 0.23)
Paraguay	439 (353 to 533)	5.44 (3.26 to 8.20)	128 (90 to 173)	1.35 (1.32 to 1.38)	-0.21 (-0.46 to 0.03)	0.24 (0.05 to 0.43)
Peru	303 (243 to 373)	2.40 (1.35 to 3.88)	74.2 (50 to 105)	1.09 (1.08 to 1.10)	-0.57 (-1.16 to 0.03)	-0.06 (-0.49 to 0.39)
Philippines	159 (125 to 197)	8.48 (6.36 to 13.5)	145 (112 to 229)	0.75 (0.74 to 0.76)	-0.95 (-1.31 to -0.59)	-0.27 (-0.59 to 0.04)
Poland	499 (399 to 616)	3.78 (2.59 to 6.44)	117 (89 to 160)	1.73 (1.62 to 1.85)	-3.05 (-3.42 to -2.68)	-1.80 (-1.96 to -1.64)
Portugal	1153 (932 to 1413)	1.36 (0.842 to 2.29)	112 (76 to 158)	2.73 (2.65 to 2.80)	-3.38 (-3.65 to -3.11)	0.27 (0.09 to 0.45)
Puerto Rico	535 (427 to 663)	16.5 (9.49 to 25.3)	305 (200 to 439)	0.83 (0.78 to 0.88)	-0.91 (-1.32 to -0.49)	-0.73 (-1.12 to -0.34)
Qatar	731 (585 to 898)	9.74 (5.11 to 15.6)	199 (129 to 283)	2.73 (2.66 to 2.80)	-0.9 (-2.11 to 0.32)	-0.51 (-1.47 to 0.45)
Republic of Korea	510 (421 to 616)	1.66 (1.06 to 2.44)	68 (49 to 93)	0.82 (0.80 to 0.84)	-4.31 (-4.44 to -4.18)	-2.29 (-2.43 to -2.15)
Republic of	497 (400 to 613)	0.455 (0.271 to 0.974)	58 (38 to 84)	1.51 (1.48 to 1.55)	-2.65 (-3.34 to -1.95)	0.39 (0.20 to 0.58)

Moldova						
Romania	489 (394 to 604)	1.44 (0.825 to 3.15)	76 (52 to 112)	1.96 (1.89 to 2.03)	-2.68 (-3.01 to -2.35)	-0.51 (-0.76 to -0.26)
Russian Federation	655 (523 to 809)	1.07 (0.597 to 3.71)	84 (56 to 136)	1.17 (1.12 to 1.21)	-0.44 (-2.02 to 1.15)	0.73 (0.54 to 0.93)
Rwanda	345 (272 to 430)	7.21 (3.81 to 11.6)	156 (99 to 231)	0.84 (0.81 to 0.86)	-1.01 (-1.20 to -0.81)	-0.83 (-1.01 to -0.65)
Saint Kitts and Nevis	334 (269 to 410)	13.7 (8.8 to 22.3)	258 (177 to 396)	1.39 (1.37 to 1.40)	-1.95 (-2.55 to -1.34)	-1.66 (-2.37 to -0.94)
Saint Lucia	319 (254 to 393)	9.77 (6.23 to 16.4)	186 (128 to 288)	1.56 (1.55 to 1.58)	-1.54 (-2.03 to -1.04)	-1.20 (-1.63 to -0.76)
Saint Vincent and the Grenadines	302 (241 to 373)	14.2 (9.33 to 22.5)	257 (179 to 384)	1.51 (1.50 to 1.52)	-1.11 (-1.29 to -0.93)	-0.85 (-1.05 to -0.64)
Samoa	168 (133 to 207)	6.63 (4 to 10.6)	123 (81 to 188)	1.07 (1.06 to 1.08)	-1.04 (-1.11 to -0.96)	-0.90 (-0.98 to -0.83)
San Marino	1129 (911 to 1381)	2.44 (1.29 to 4.11)	124 (86 to 173)	1.74 (1.69 to 1.78)	-1.63 (-1.78 to -1.48)	0.37 (0.32 to 0.42)
Sao Tome and Principe	377 (297 to 469)	9.51 (4.69 to 16.6)	202 (117 to 325)	0.98 (0.98 to 0.99)	0.62 (0.47 to 0.78)	0.66 (0.53 to 0.79)
Saudi Arabia	1172 (946 to 1446)	5.9 (3.3 to 9.31)	177 (124 to 244)	3.08 (3.02 to 3.15)	-1.04 (-1.17 to -0.92)	0.34 (0.28 to 0.40)
Senegal	313 (247 to 388)	8.07 (3.72 to 14.8)	170 (94 to 287)	0.56 (0.54 to 0.58)	-0.66 (-1.13 to -0.19)	-0.49 (-0.94 to -0.04)
Serbia	652 (522 to 798)	7.44 (4.79 to 11)	191 (139 to 258)	1.94 (1.91 to 1.96)	-1.99 (-2.34 to -1.64)	-1.21 (-1.47 to -0.95)
Seychelles	156 (125 to 193)	11.7 (7.41 to 17.3)	201 (134 to 286)	1.32 (1.31 to 1.33)	-1.37 (-1.56 to -1.17)	-1.29 (-1.61 to -0.96)
Sierra Leone	300 (238 to 373)	6.98 (3.19 to 12.3)	151 (85 to 243)	0.53 (0.51 to 0.55)	-0.66 (-0.76 to -0.55)	-0.45 (-0.53 to -0.37)
Singapore	488 (395 to 592)	0.222 (0.125 to 0.423)	46 (29 – 68)	0.52 (0.50 to 0.53)	-7.26 (-7.92 to -6.59)	-1.44 (-1.59 to -1.29)
Slovakia	547 (438 to 678)	3.74 (2.32 to 5.78)	119 (85 to 162)	2.30 (2.26 to 2.34)	-2.26 (-2.67 to -1.85)	-1.00 (-1.30 to -0.69)
Slovenia	596 (481 to 733)	1.85 (1.01 to 4.14)	88 (60 to 132)	2.24 (2.20 to 2.29)	-4.85 (-5.30 to -4.39)	-2.06 (-2.46 to -1.65)
Solomon Islands	136 (108 to 168)	7.43 (3.98 to 13.8)	142 (82 to 252)	0.94 (0.92 to 0.96)	-0.33 (-0.45 to -0.21)	-0.33 (-0.47 to -0.19)
Somalia	294 (230 to 369)	8.08 (3.67 to 14.1)	172 (94 to 276)	0.36 (0.34 to 0.38)	-0.37 (-0.47 to -0.28)	-0.30 (-0.39 to -0.21)
South Africa	451 (355 to 563)	8.30 (6.68 to 10.4)	176 (147 to 216)	0.64 (0.62 to 0.66)	0.49 (-0.18 to 1.17)	0.45 (-0.04 to 0.95)
South Sudan	286 (226 to 358)	6.59 (2.75 to 12.4)	140 (75 to 236)	0.53 (0.50 to 0.56)	-0.11 (-0.18 to -0.05)	-0.08 (-0.14 to -0.02)
Spain	1324 (1073 to 1633)	0.761 (0.453 to 1.61)	126 (81 to 186)	2.73 (2.64 to 2.82)	-4.18 (-4.49 to -3.86)	1.07 (0.93 to 1.21)
Sri Lanka	156 (125 to 191)	14.1 (8.11 to 22.2)	229 (143 to 349)	1.25 (1.22 to 1.28)	0.47 (0.23 to 0.71)	0.33 (0.06 to 0.61)
Sudan	482 (386 to 587)	6.24 (2.48 to 11.3)	137 (76 to 218)	2.55 (2.52 to 2.59)	-0.55 (-0.66 to -0.43)	-0.02 (-0.08 to 0.03)
Suriname	285 (229 to 352)	6.33 (4.02 to 9.56)	134 (94 to 188)	1.43 (1.42 to 1.44)	-0.97 (-1.36 to -0.58)	-0.61 (-1.00 to -0.23)
Sweden	1208 (967 to 1486)	2.11 (1.42 to 3.68)	118 (85 to 161)	0.45 (0.39 to 0.50)	-1.30 (-1.54 to -1.05)	-0.22 (-0.34 to -0.10)
Switzerland	1200 (972 to 1470)	0.718 (0.43 to 1.41)	105 (69 to 154)	1.85 (1.81 to 1.89)	-4.74 (-5.29 to -4.19)	0.04 (-0.09 to 0.16)
Syrian Arab Republic	640 (512 to 782)	17.6 (8.94 to 28.3)	304 (185 to 452)	2.67 (2.59 to 2.75)	-0.72 (-1.09 to -0.35)	-0.39 (-0.70 to -0.09)
Taiwan (Province	309 (259 to 373)	10.4 (6.32 to 16.9)	196 (131 to 292)	2.71 (2.63 to 2.79)	-1.71 (-2.05 to -1.37)	-1.49 (-1.73 to -1.24)

of China)						
Tajikistan	466 (372 to 577)	8.28 (3.68 to 12.9)	207 (113 to 300)	1.07 (1.06 to 1.09)	3.59 (3.23 to 3.95)	2.80 (2.53 to 3.07)
Thailand	146 (117 to 179)	6.66 (3.85 to 10.4)	124 (77 to 185)	1.25 (1.25 to 1.26)	-1.30 (-1.84 to -0.76)	-1.17 (-1.70 to -0.64)
Timor-Leste	124 (98.9 to 153)	8.83 (4.13 to 16.9)	151 (77 to 281)	1.16 (1.15 to 1.17)	-0.29 (-0.36 to -0.23)	-0.26 (-0.35 to -0.18)
Togo	338 (267 to 421)	7.36 (3.60 to 12.8)	162 (94 to 256)	0.70 (0.69 to 0.71)	-0.72 (-0.98 to -0.46)	-0.45 (-0.67 to -0.23)
Tokelau	168 (134 to 208)	5.87 (3.45 to 9.42)	110 (71 to 167)	0.97 (0.88 to 1.06)	-1.12 (-1.17 to -1.07)	-0.99 (-1.04 to -0.94)
Tonga	178 (142 to 220)	6.13 (3.54 to 10.4)	118 (75 to 190)	1.03 (1.02 to 1.04)	-0.33 (-0.70 to 0.05)	-0.20 (-0.53 to 0.12)
Trinidad and Tobago	338 (271 to 414)	10.2 (5.65 to 21.5)	209 (130 to 397)	1.41 (1.39 to 1.43)	-1.79 (-2.11 to -1.47)	-1.50 (-1.78 to -1.23)
Tunisia	638 (510 to 779)	5.33 (3.00 to 8.97)	137 (94 to 197)	3.09 (3.06 to 3.12)	-0.7 (-0.81 to -0.59)	0.31 (0.18 to 0.44)
Turkey	612 (489 to 750)	7.78 (4.39 to 11.8)	172 (118 to 232)	2.55 (2.52 to 2.57)	-1.52 (-1.9 to -1.15)	-0.75 (-1.00 to -0.51)
Turkmenistan	469 (377 to 579)	4.23 (2.56 to 6.58)	131 (90 to 186)	1.15 (1.11 to 1.18)	1.42 (0.41 to 2.45)	1.33 (0.62 to 2.04)
Tuvalu	162 (129 to 200)	7.55 (4.39 to 12.3)	138 (87 to 214)	1.01 (0.99 to 1.02)	-0.89 (-0.95 to -0.83)	-0.89 (-0.96 to -0.82)
Uganda	339 (268 to 421)	6.43 (3.48 to 10.4)	143 (92 to 211)	0.80 (0.79 to 0.82)	-0.21 (-0.29 to -0.13)	-0.05 (-0.11 to 0.02)
Ukraine	503 (402 to 621)	0.608 (0.364 to 1.29)	62 (42 to 86)	1.39 (1.37 to 1.4)	-2.17 (-2.64 to -1.71)	0.33 (0.12 to 0.53)
United Arab Emirates	954 (767 to 1170)	14.0 (6.97 – 26.0)	287 (177 to 461)	3.61 (3.49 to 3.72)	-2.04 (-2.71 to -1.37)	-1.30 (-1.89 to -0.71)
United Kingdom	1094 (858 to 1361)	0.95 (0.722 to 1.73)	91 (63 to 127)	1.49 (1.41 to 1.58)	-2.83 (-3.15 to -2.5)	0.26 (0.06 to 0.46)
United Republic of Tanzania	344 (271 to 428)	6.78 (3.45 to 11.4)	150 (92 to 229)	0.72 (0.71 to 0.73)	-0.33 (-0.52 to -0.14)	-0.14 (-0.24 to -0.05)
United States of America	1195 (956 to 1488)	2.71 (1.76 to 3.75)	145 (107 to 189)	0.51 (0.42 to 0.60)	-0.74 (-1.04 to -0.44)	0.06 (-0.11 to 0.22)
United States Virgin Islands	480 (385 to 591)	11.6 (7.40 to 17.2)	235 (166 to 324)	1.27 (1.18 to 1.36)	-0.73 (-0.92 to -0.54)	-0.54 (-0.79 to -0.28)
Uruguay	919 (739 to 1125)	2.82 (1.80 to 4.41)	126 (90 to 172)	0.50 (0.46 to 0.54)	-1.36 (-1.83 to -0.88)	-0.35 (-0.48 to -0.23)
Uzbekistan	501 (402 to 619)	9.78 (6.07 to 14.9)	242 (163 to 344)	1.19 (1.17 to 1.21)	4.71 (4.33 to 5.10)	3.60 (3.18 to 4.02)
Vanuatu	139 (110 to 172)	8.21 (4.70 to 13.5)	148 (91 to 233)	0.84 (0.83 to 0.85)	-0.26 (-0.37 to -0.15)	-0.19 (-0.33 to -0.05)
Venezuela (Bolivarian Republic of)	201 (161 to 248)	2.33 (1.23 to 5.36)	62 (39 to 115)	0.83 (0.80 to 0.86)	-1.96 (-2.65 to -1.27)	-1.36 (-1.98 to -0.73)
Viet Nam	136 (109 to 167)	12.7 (6.14 to 27.3)	202 (106 to 425)	1.07 (1.06 to 1.07)	-0.66 (-0.71 to -0.61)	-0.72 (-0.78 to -0.66)
Yemen	467 (373 to 576)	6.35 (2.43 to 11.4)	143 (77 to 225)	2.03 (2.01 to 2.06)	-0.55 (-0.65 to -0.46)	-0.06 (-0.14 to 0.03)
Zambia	365 (289 to 452)	5.99 (3.42 to 9.64)	139 (92 to 201)	0.91 (0.90 to 0.92)	-1.01 (-1.11 to -0.91)	-0.64 (-0.72 to -0.56)
Zimbabwe	371 (294 to 461)	7.08 (4.16 to 11.1)	158 (105 to 228)	0.49 (0.45 to 0.53)	0.84 (0.67 to 1.01)	0.75 (0.56 to 0.95)

Note: Estimates are for individuals aged over 65 years. AAPCs=average annual percent changes. CI=confidence interval. DALYs=disability-adjusted life years. P=P value for the significant test of AAPCs. Numbers in parentheses are 95% uncertainty intervals (Age-standardized rate) and 95% confidence interval (AAPCs).

Supplementary Figure 1. The changes in the proportion of prevalence cases among type 1 diabetes mellitus patients (T1DM) aged over 65 years to the overall T1DM patients from 1990 to 2019.

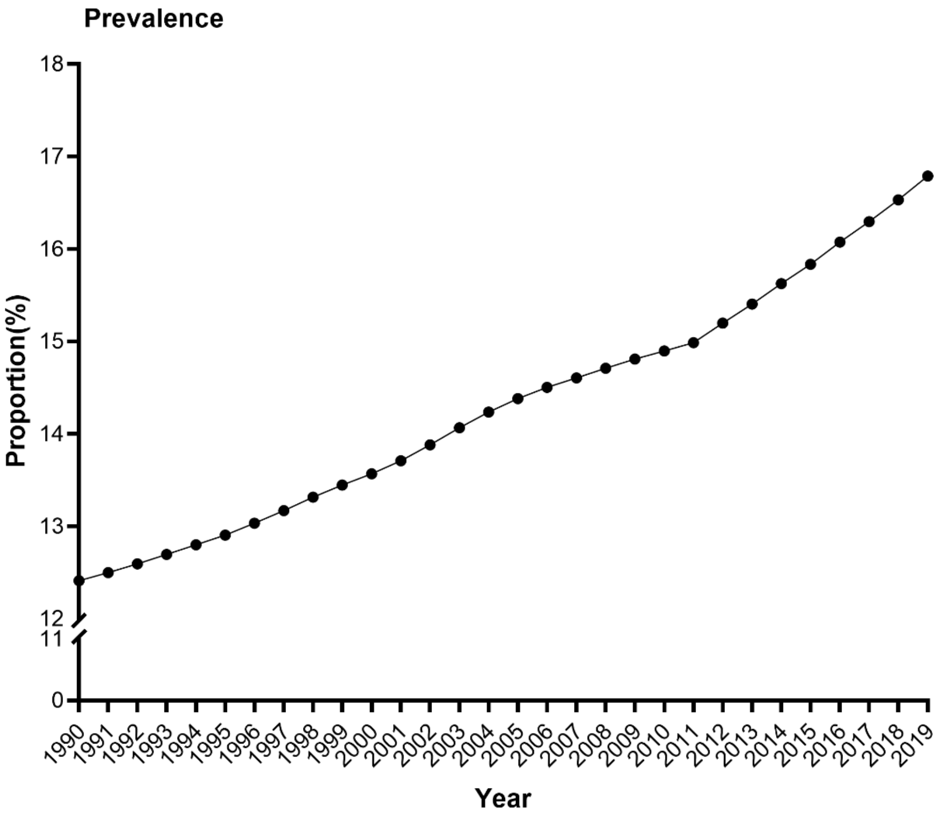

Supplementary Figure 2. Temporal trend of age-standardized prevalence (A) mortality (B) and disability-adjusted life years (C) for type 1 diabetes mellitus patients aged over 65 years and overall T1DM patients from 1990 to 2019.

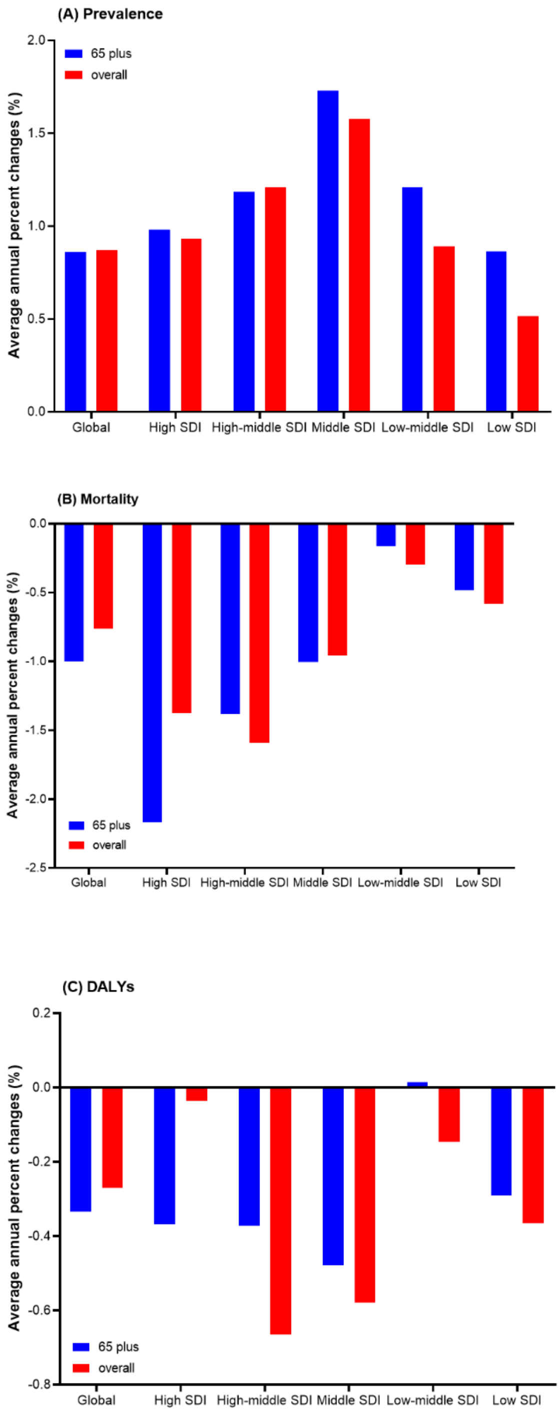

Supplementary Figure 3. The age-standardized mortality (A) and disability-adjusted life years (B) proportion of type 1 diabetes mellitus to all-cause in patients aged over 65 years from 1990 to 2019.

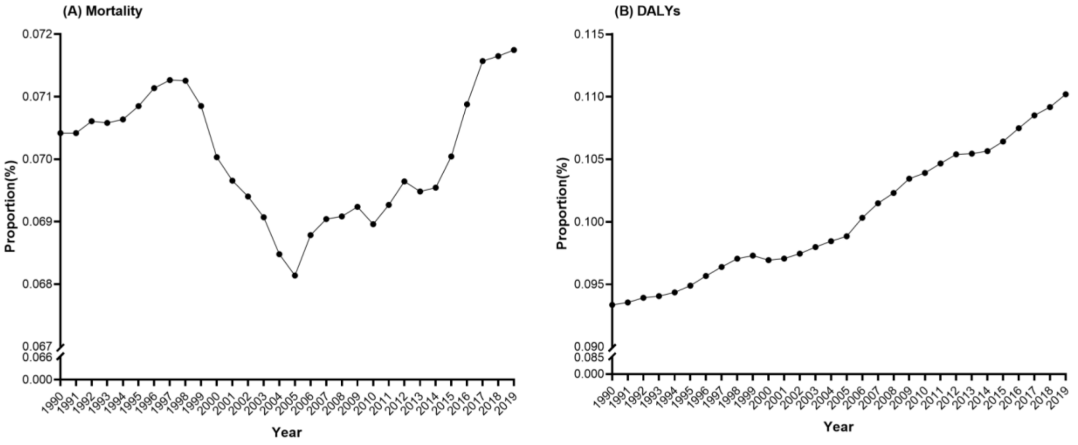

Supplementary Figure 4. Temporal trend of age-standardized mortality and disability-adjusted life years of type 1 diabetes mellitus in elderly people from 1990 to 2019 at global and socio-demographic index levels by sex.

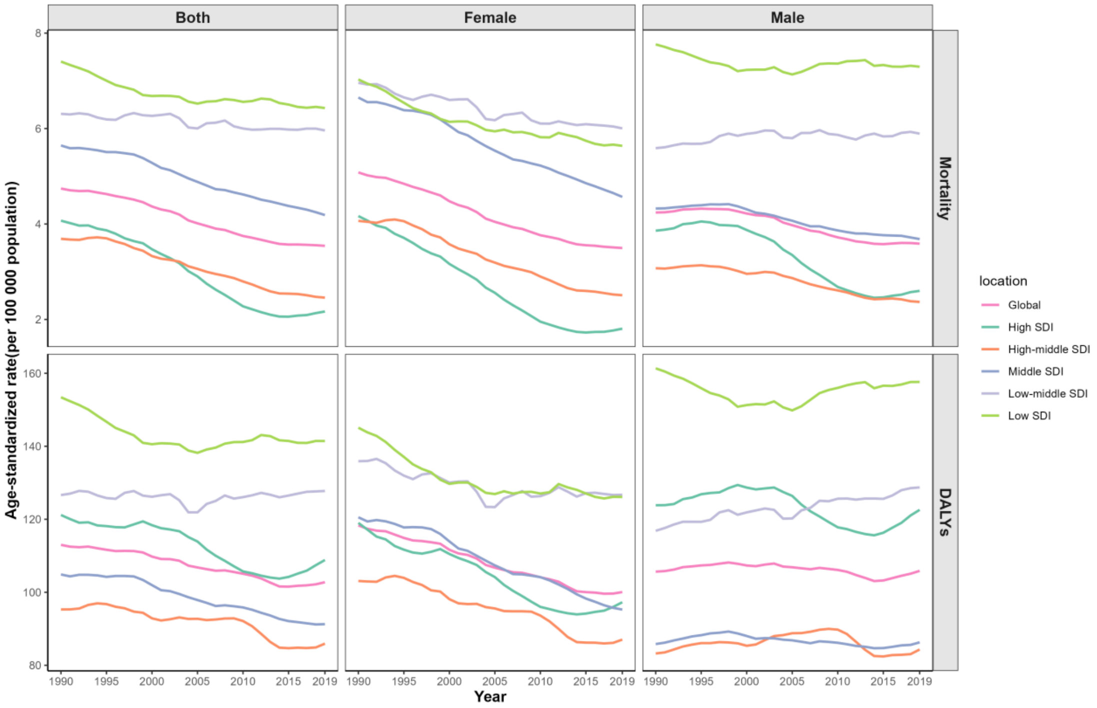

Supplementary Figure 5. Average annual percent changes of age-standardized mortality and disability-adjusted life years of type 1 diabetes mellitus in elderly people aged over 65 years from 1990 to 2019 at socio-demographic index levels by sex.

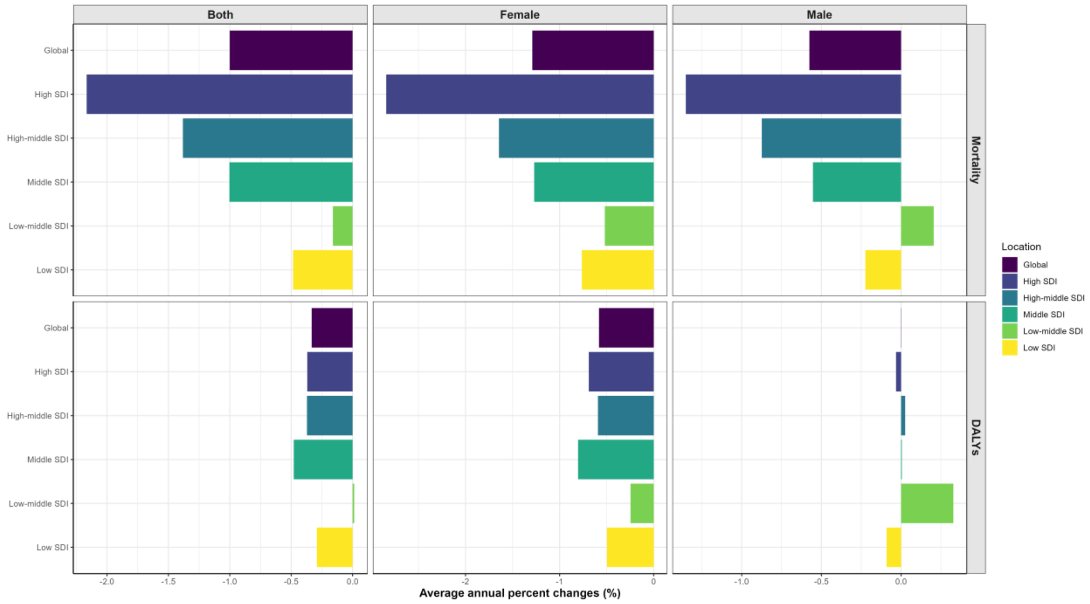

Supplementary Figure 6. Average annual percent changes of age-standardized prevalence, mortality and disability-adjusted life years of type 1 diabetes mellitus in elderly people from 1990 to 2019 by sex and age.

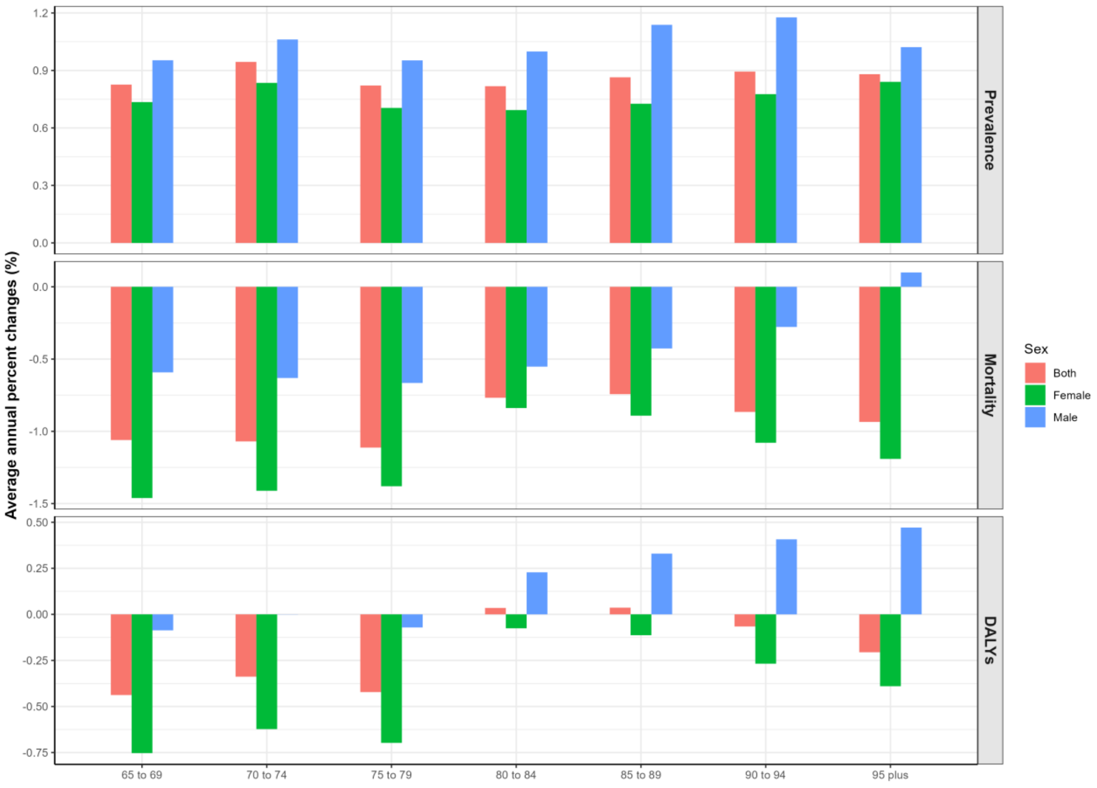

Supplementary Figure 7. Temporal trend of age-standardized prevalence, mortality and disability-adjusted life years of type 1 diabetes mellitus aged over 65 years and overall T1DM patients from 1990 to 2019 at global and socio-demographic index levels.

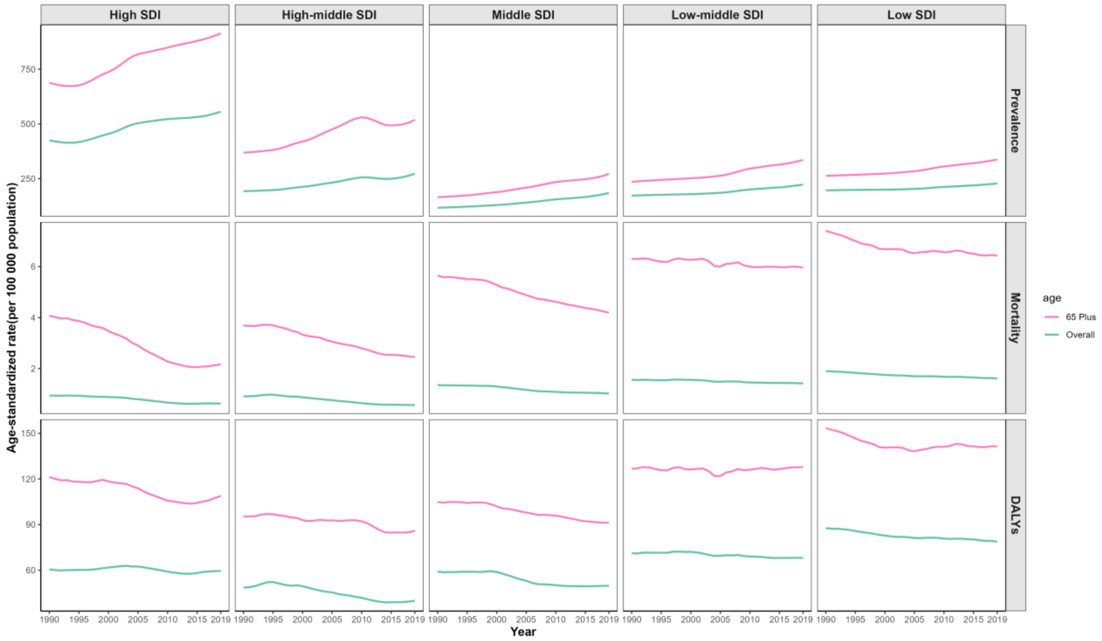

Supplementary Figure 8. Average annual percent changes of age-standardized prevalence (A), mortality (B) and disability-adjusted life years (C) of type 1 diabetes mellitus aged over 65 years and overall T1DM patients from 1990 to 2019 at global and socio-demographic index levels.

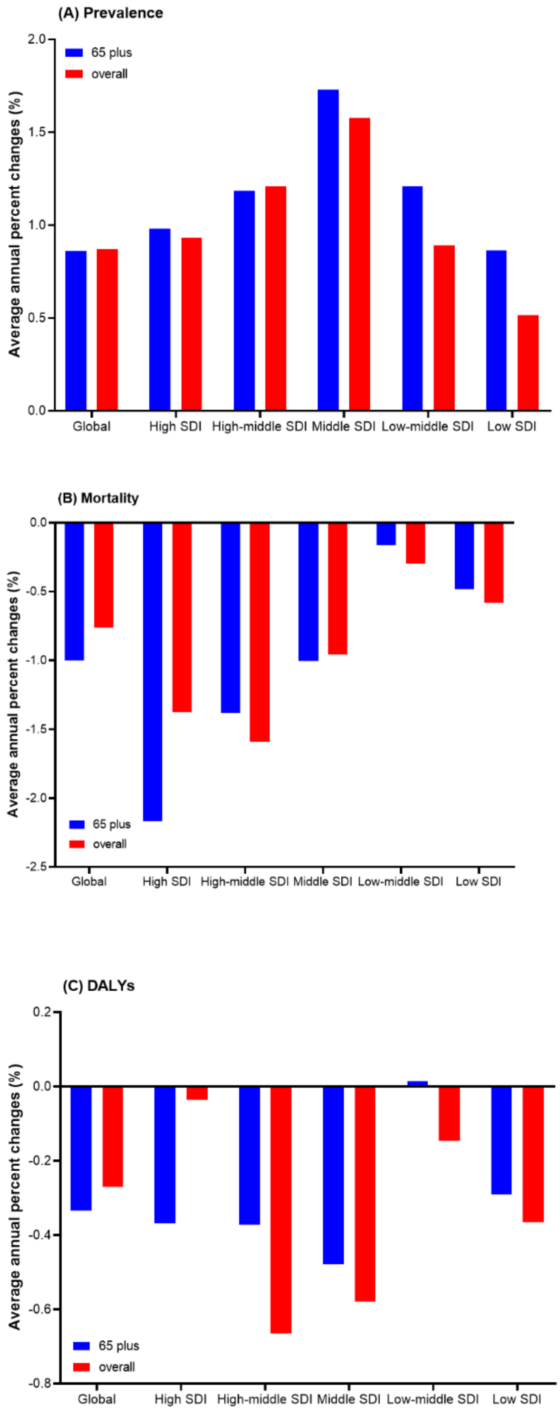

Supplementary Figure 9. Prevalence (A), mortality (B) and disability-adjusted life years (C) rate of type 1 diabetes mellitus in elderly people from 204 countries according to the socio-demographic index in 2019.

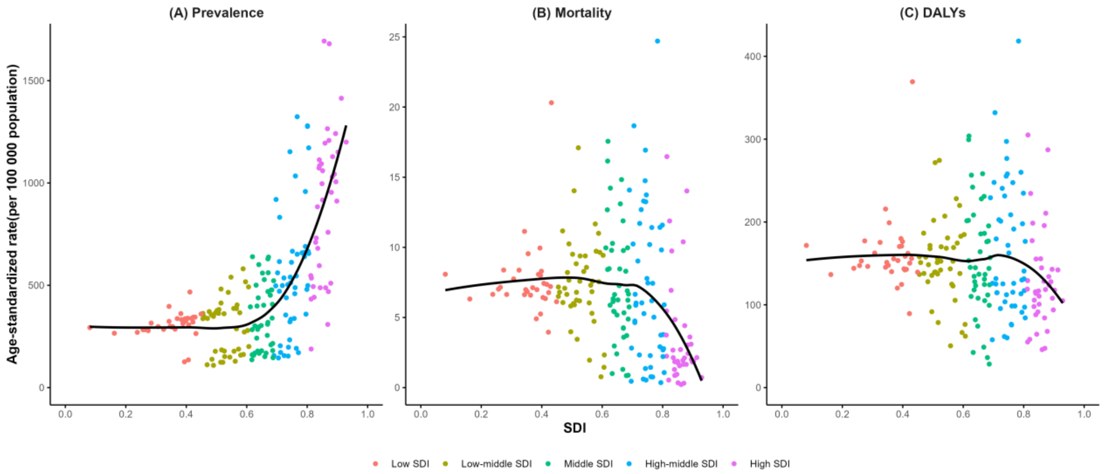

Supplementary Figure 10. Average annual percent changes of age-standardized prevalence, mortality and disability-adjusted life years of type 1 diabetes mellitus in elderly people aged over 65 years from 1990 to 2019 at regions levels.

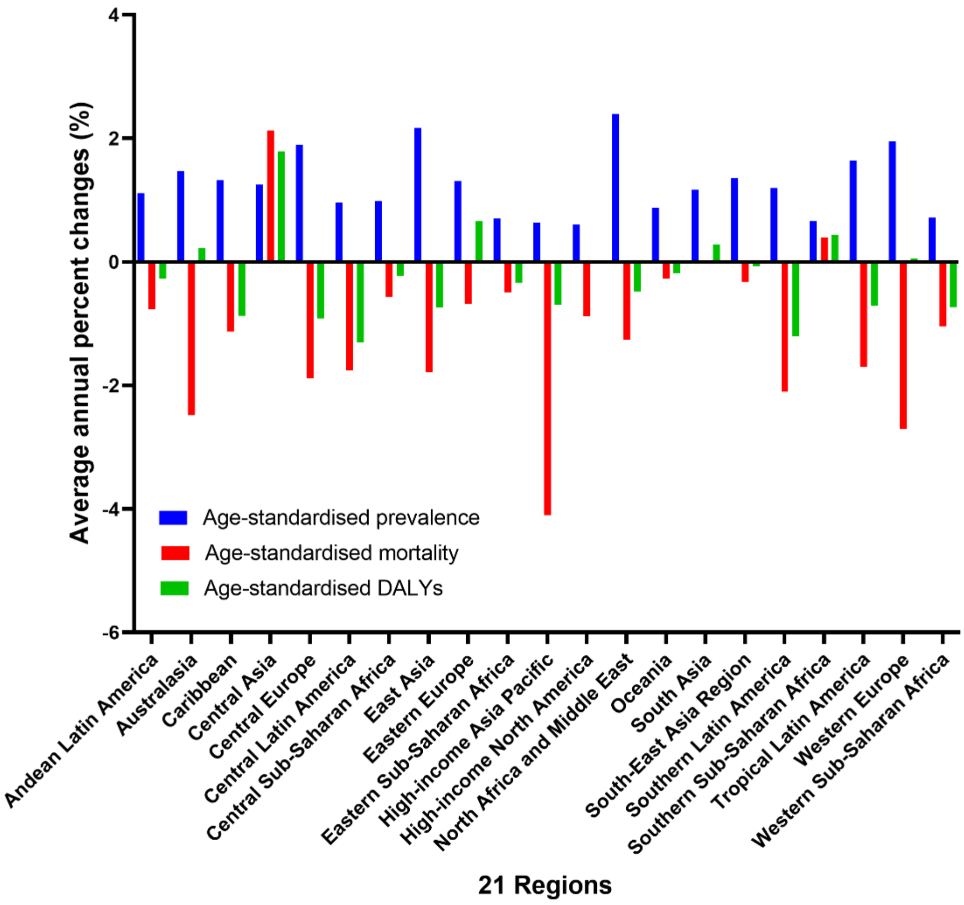

Supplementary Figure 11. Average annual percent changes of age-standardized mortality and disability-adjusted life years of type 1 diabetes mellitus in elderly people aged over 65 years from 1990 to 2019 at regions levels by sex.

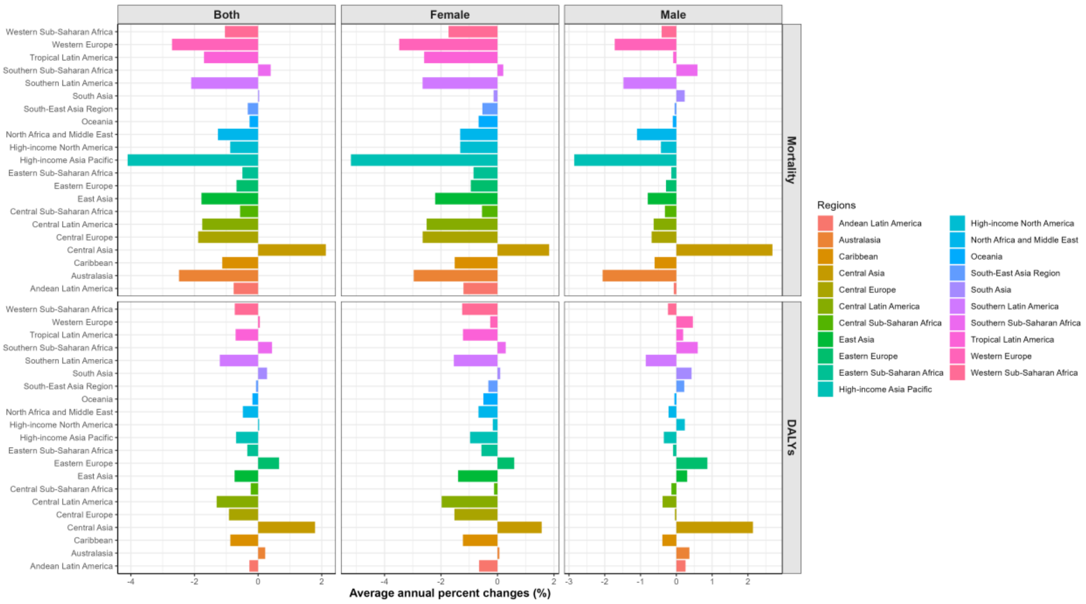

STROBE Statement—Checklist of items that should be included in reports of the current study

	Item No	Recommendation	Page No
Title and abstract	1	(*a*) Indicate the study's design with a commonly used term in the title or the abstract	1-3
		(*b*) Provide in the abstract an informative and balanced summary of what was done and what was found	2-4
**Introduction**			
Background/rationale	2	Explain the scientific background and rationale for the investigation being reported	5
Objectives	3	State specific objectives, including any prespecified hypotheses	5-6
**Methods**			
Study design	4	Present key elements of study design early in the paper	6-9
Setting	5	Describe the setting, locations, and relevant dates, including periods of recruitment, exposure, follow-up, and data collection	6-9
Participants	6	(*a*) Give the eligibility criteria, and the sources and methods of selection of participants	6-9
Variables	7	Clearly define all outcomes, exposures, predictors, potential confounders, and effect modifiers. Give diagnostic criteria, if applicable	6-9
Data sources/measurement	8*	For each variable of interest, give sources of data and details of methods of assessment (measurement). Describe comparability of assessment methods if there is more than one group	6-9
Bias	9	Describe any efforts to address potential sources of bias	6-9
Study size	10	Explain how the study size was arrived at	N/A
Quantitative variables	11	Explain how quantitative variables were handled in the analyses. If applicable, describe	6-9

		which groupings were chosen and why	
Statistical methods	12	(*a*) Describe all statistical methods, including those used to control for confounding	6-9
		(*b*) Describe any methods used to examine subgroups and interactions	6-9
		(*c*) Explain how missing data were addressed	Supplementary Methods
		(*d*) If applicable, describe analytical methods taking account of sampling strategy	6-9
		(*e*) Describe any sensitivity analyses	N/A
**Results**			
Participants	13*	(a) Report numbers of individuals at each stage of study—eg numbers potentially eligible, examined for eligibility, confirmed eligible, included in the study, completing follow-up, and analysed	9-14
		(b) Give reasons for non-participation at each stage	Supplementary Methods
		(c) Consider use of a flow diagram	Supplementary Methods
Descriptive data	14*	(a) Give characteristics of study participants (eg demographic, clinical, social) and information on exposures and potential confounders	9-14
		(b) Indicate number of participants with missing data for each variable of interest	Supplementary Methods
Outcome data	15*	Report numbers of outcome events or summary measures	9-14
Main results	16	(*a*) Give unadjusted estimates and, if applicable, confounder-adjusted estimates and their precision (eg, 95% confidence interval). Make clear which confounders were adjusted for and why they were included	9-14
		(*b*) Report category boundaries when continuous variables were categorized	9-14
		(*c*) If relevant, consider translating estimates of relative	N/A

		risk into absolute risk for a meaningful time period	
Other analyses	17	Report other analyses done—eg analyses of subgroups and interactions, and sensitivity analyses	Supplementary Tables 1-5 and Supplementary Figures 1-11
**Discussion**			
Key results	18	Summarise key results with reference to study objectives	14
Limitations	19	Discuss limitations of the study, taking into account sources of potential bias or imprecision. Discuss both direction and magnitude of any potential bias	18-19
Interpretation	20	Give a cautious overall interpretation of results considering objectives, limitations, multiplicity of analyses, results from similar studies, and other relevant evidence	14-19
Generalisability	21	Discuss the generalisability (external validity) of the study results	19
**Other information**			
Funding	22	Give the source of funding and the role of the funders for the present study and, if applicable, for the original study on which the present article is based	20

*Give information separately for exposed and unexposed groups.

Note: An Explanation and Elaboration article discusses each checklist item and gives methodological background and published examples of transparent reporting. The STROBE checklist is best used in conjunction with this article (freely available on the Web sites of PLoS Medicine at http://www.plosmedicine.org/, Annals of Internal Medicine at http://www.annals.org/, and Epidemiology at http://www.epidem.com/). Information on the STROBE Initiative is available at www.strobe-statement.org.
